# Discovery of ALA-hydroxypyridinone hybrids: novel strategies for photodynamic therapy against superficial tumours

**DOI:** 10.1080/14756366.2026.2700840

**Published:** 2026-07-08

**Authors:** Xiaoying Jiang, Meiling Feng, Xiaotian Niu, Shan Wang, Yinyan Sun, Wenchao Chen, Renren Bai

**Affiliations:** ^a^ School of Pharmacy, Hangzhou Normal University, Hangzhou, PR China; ^b^ Key Laboratory of Elemene Class Anti-Cancer Chinese Medicines; Engineering Laboratory of Development and Application of Traditional Chinese Medicines; Collaborative Innovation Center of Traditional Chinese Medicines of Zhejiang Province, Hangzhou Normal University, Hangzhou, PR China

**Keywords:** Photodynamic therapy (PDT), photosensitiser, iron chelator, 5-aminolevulinic acid (ALA), hydroxypyridinone (HPO)

## Abstract

Superficial tumours can be effectively treated with photodynamic therapy (PDT), which is characterised by its targeted approach and enhanced safety profile. 5-aminolevulinic acid (ALA), a precursor to the endogenous photosensitiser PpIX, has been widely utilised in PDT. However, its high hydrophilicity, poor permeability and stability, necessitates structural modifications to improve its drug-like properties and increase the PpIX yield. Hydroxypyridinone (HPO) iron chelators can synergistically enhance PpIX production when administered in conjunction with ALA, as well as inducing tumour cell death through iron chelation. Consequently, this study designed a series of novel ALA-HPO derivatives, functioning as dual prodrugs to elevate intracellular PpIX levels and enhance photodynamic anti-tumour effects. The optimised compound **AP-8** displayed the highest phototoxicity and greatest PpIX yield, demonstrating superior photodynamic anti-tumour efficacy compared to ALA in a mouse melanoma model, with a TGI of 84.74%. These findings underscore the potent application potential of ALA-HPO derivatives in PDT.

## GRAPHICAL ABSTRACT

**Figure:**
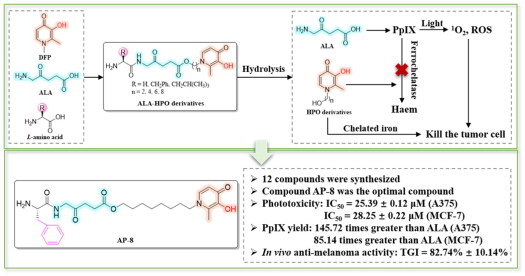

## Introduction

The treatment options for conventional tumours primarily encompass chemotherapy, radiotherapy, immunotherapy, and surgical intervention. In contrast, superficial tumours – including superficial skin cancers (e.g. melanoma, basal cell carcinoma, and squamous cell carcinoma), superficial breast cancer, lipomas, haemangiomas, lymphomas, and neurofibromas – can be treated effectively with photodynamic therapy (PDT)^1–4^. PDT is distinguished by its precise temporal and spatial control, minimally invasive nature, targeted delivery, enhanced selectivity and safety, and high efficiency^5^^,^^6^.

**Figure 1.**
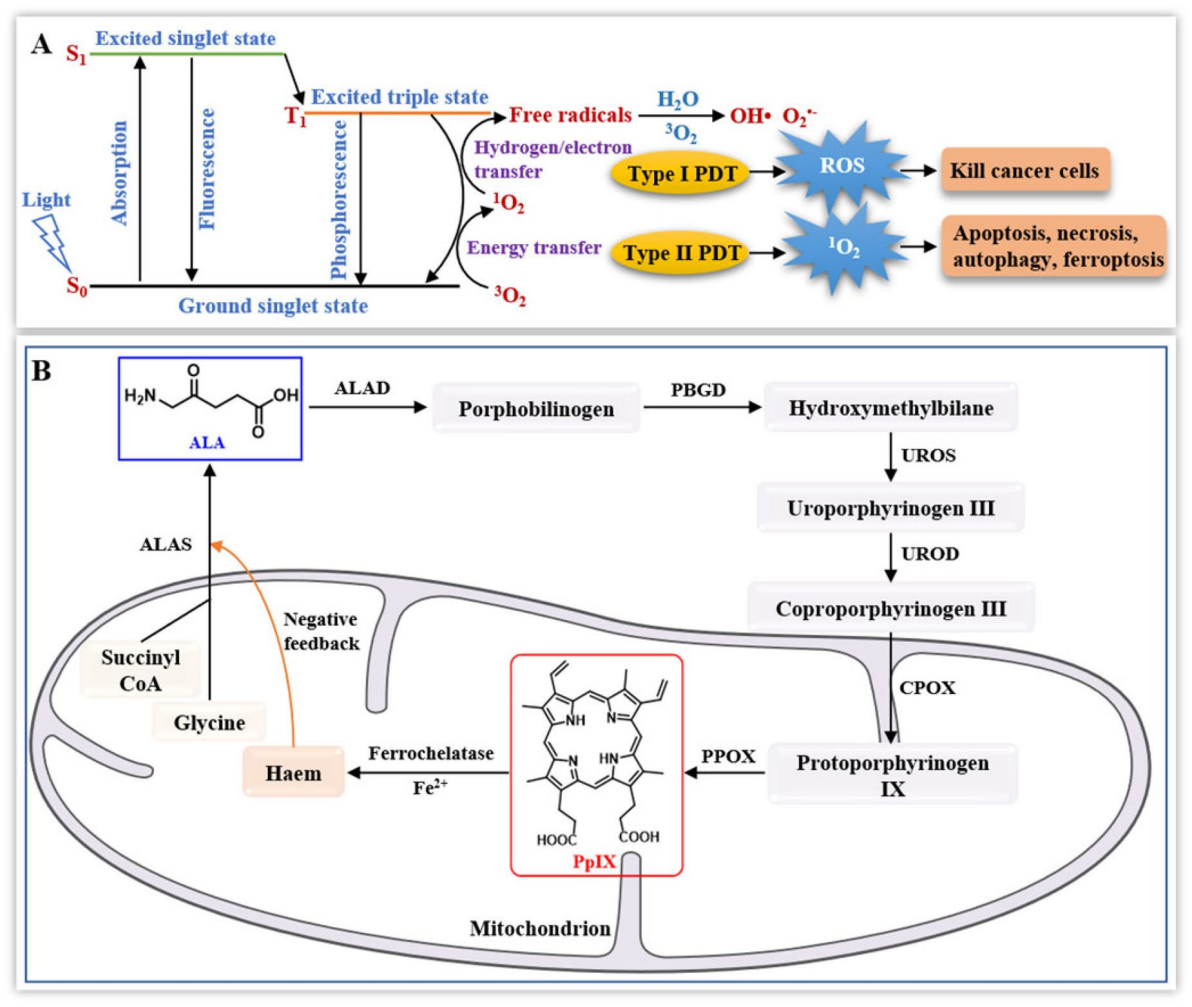
(A) The mechanism of PDT; (B) The haem biosynthesis pathway. ALAD: ALA dehydratase; PBGD: porphobilinogen deaminase; UROS: uroporphyrinogen-III synthase; UROD: uroporphyrinogen decarboxylase; CPOX: coproporphyrinogen oxidase; PPOX: protoporphyrinogen oxidase; ALAS: ALA synthase.

The mechanism of PDT relies on the interaction between a photosensitiser, specific wavelengths of light, and oxygen, with the photosensitiser serving a crucial role. When exposed to a light source that aligns with its absorption spectrum, the photosensitiser transitions from the ground state (S_0_) to the excited singlet state (S_1_). The S_1_ state is unstable (nanosecond timescale) and can decay back to the S_0_ state through either emission (fluorescence) or thermal dissipation^7^. Additionally, it can undergo intersystem crossing to generate a longer-lived (microsecond timescale) and more stable excited triplet state (T_1_)^8^. In the T_1_ state, the photosensitiser can participate in Type I and Type II reactions, or it may release energy *via* phosphorescent emissions through radiative transitions (Figure 1(A)).

In the Type I reaction, the photosensitiser in the T_1_ state directly interacts with biological substrates through hydrogen or electron transfer, resulting in the formation of free radicals and radical ions. These free radicals subsequently react with water or triplet oxygen (^3^O_2_) to generate hydroxyl radicals (OH·), hydrogen peroxide (H_2_O_2_), or superoxide anions (O_2_·^−^). This cascade of reactions leads to the production of reactive oxygen species (ROS), which induces oxidative stress and ultimately results in tumour cell death^9^.

In the Type II reaction, the photosensitiser in the T_1_ state directly transfers energy to ground-state triplet oxygen (^3^O_2_), converting it into the cytotoxic singlet oxygen (^1^O_2_)^10^. ^1^O_2_ exhibits strong oxidising properties, leading to oxidative damage to various biological substrates and resulting in apoptosis, necrosis, autophagy, or ferroptosis in tumour cells^11^^,^^12^. The Type II reaction plays a predominant role in PDT, although its effectiveness is significantly influenced by oxygen concentration. Type II reaction is optimised in oxygen-rich environments, whereas Type I reaction predominantly occurs in hypoxic conditions^11^.

5-aminolevulinic acid (Levulan^®^, ALA) is a small molecule photosensitiser commonly used in clinical practice, classified as a second-generation porphyrin-type photosensitiser. Unlike other porphyrin photosensitizers, ALA is not intrinsically photosensitive; rather, it is a natural, endogenous and hydrophilic prodrug that must be converted into the photosensitising agent protoporphyrin IX (PpIX) *via* the haem synthesis pathway within cells. Upon light excitation, PpIX produces ^1^O_2_ and ROS, which induce cytotoxic effects and lead to the destruction of tumour cells. However, PpIX will interact with ferrochelatase in the mitochondria, leading to the production of haem (Figure 1(B))^13^. Tumour or proliferative cells exhibit a selective ability to uptake ALA, with the conversion of ALA to PpIX serving as the primary rate-limiting step. By externally increasing the availability of ALA, this rate-limiting step can be circumvented, leading to a substantial production of PpIX^14^. The subsequent conversion of PpIX to haem represents a secondary rate-limiting step. Inhibiting the activity of ferrochelatase through the use of iron chelators can block this conversion, thereby further elevating PpIX concentrations within the target cells^15^. Notably, most tumour cells demonstrate increased activity of the upstream enzymes involved in PpIX synthesis, alongside reduced activity of ferrochelatase, facilitating the selective accumulation of PpIX in tumour cells and enhancing the potential for targeted therapy^16^.

**Figure 2.**
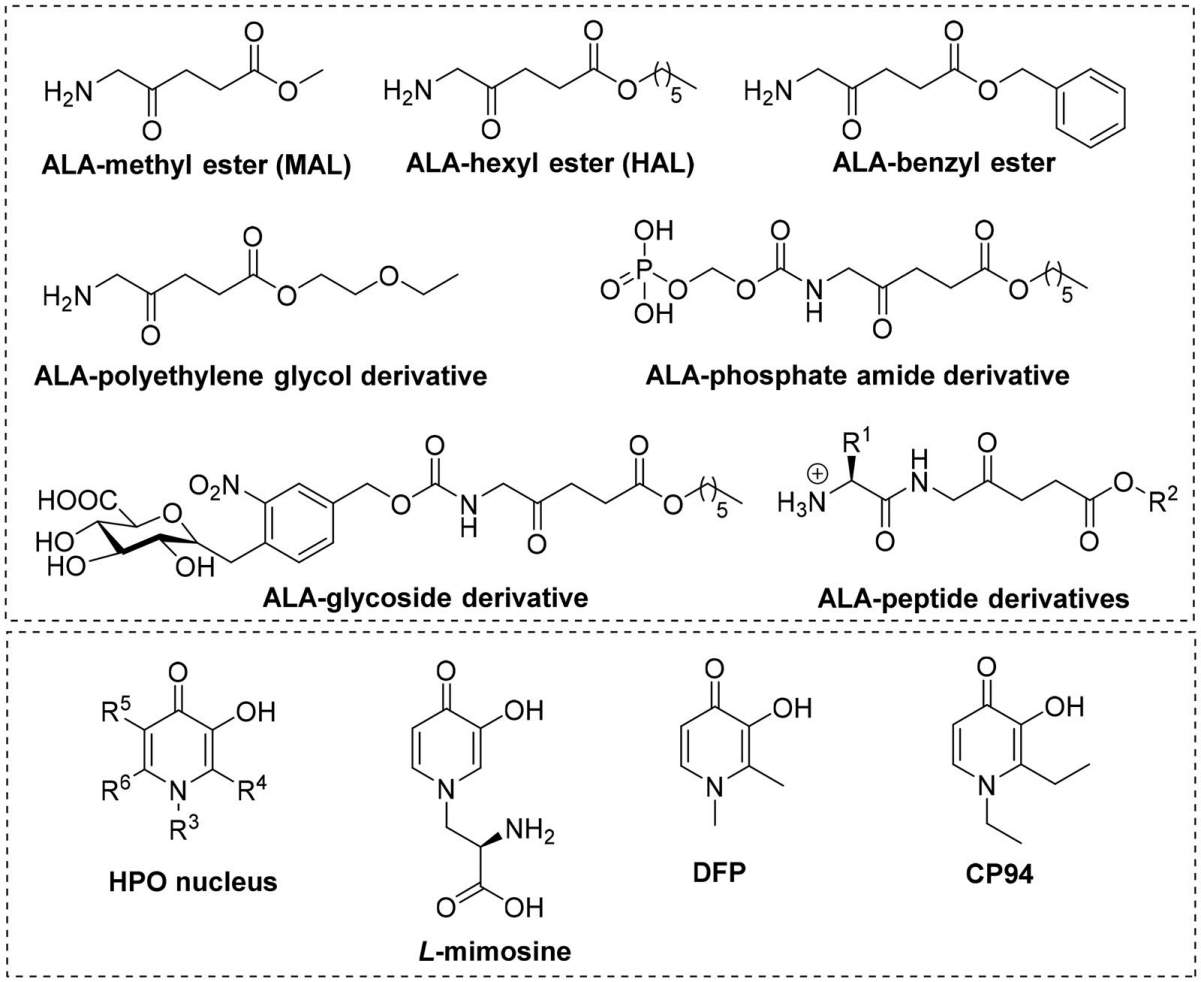
The chemical structures of ALA derivatives, HPO nucleus, *L*-mimosine, DFP and CP94.

Although ALA possesses advantages such as high solubility, straightforward metabolism, and elevated production of ROS, its amphiphilic structure leads to poor stability under physiological pH conditions, as well as low membrane permeability and bioavailability^17^. Research suggests that structural modifications to ALA can be classified into two primary categories. First, esterification modifications at the carboxyl terminal aim to enhance lipophilicity, improve cell membrane permeability, and increase intracellular production of PpIX. Examples of such modifications include the commercially approved ALA-methyl ester (MAL) and ALA-hexyl ester (HAL), along with ALA-benzyl ester and ALA-polyethylene glycol derivatives^15^^,^^18–20^. Second, amidation modifications at the amino terminal seek to improve stability and PpIX production, provided that the amide bond remains hydrolysable by intracellular enzymes. Notable derivatives in this category include ALA-phosphate amide derivatives, ALA-glycoside derivatives, and ALA-peptide derivatives, which can be hydrolysed by intracellular phosphatases, β-glucuronidase, and peptidases, respectively, facilitating the release of ALA (Figure 2)^20–22^.

Iron chelators not only target the ferrochelatase involved in the haem synthesis pathway to enhance ALA-based PDT, but they also exert antitumor effects through a variety of mechanisms. Tumour cells typically experience an imbalance in iron homeostasis, resulting in an increased demand for iron to support their metabolism and proliferation^23^. Iron chelators inhibit cell proliferation and metastasis through various mechanisms. These include depleting intracellular iron, inactivating ribonucleotide reductase, blocking DNA synthesis, altering the expression of multiple cyclins, inducing cell cycle arrest, and inhibiting several signalling pathways, such as the WNT signalling pathway^24–26^. Additionally, they impact the tumour cell microenvironment, further contributing to the suppression of tumour progression^27^.

Among the various iron chelators, hydroxypyridinone (HPO)-based chelators exhibit significant research potential due to their high affinity and selectivity for iron under physiological conditions, ease of derivatization, and favourable biocompatibility. Notably, the representative drug, Deferiprone (DFP), received FDA approval for marketing in 2011 (Figure 2)^28^. HPO-derived iron chelators have demonstrated significant antitumor effects. For instance, *L*-mimosine can inhibit malignant melanoma by enhancing the production of ROS, activating both endogenous and exogenous apoptotic pathways, and inducing cell cycle arrest^29^. Additionally, HPO-derived iron chelators, such as CP94, when combined with ALA or its derivatives, have been found to increase the accumulation of PpIX and enhance cytotoxicity^30^^,^^31^. Notably, the conjugates of ALA and HPO-derived iron chelators demonstrate higher levels of PpIX and greater antitumor efficacy than either ALA alone or the combination of CP94 with ALA^32–34^. This observation suggests that the integration of ALA with HPO-derived iron chelators not only addresses the pharmacological deficiencies of the individual agents but also synergistically enhances their antitumor activity.

Based on the aforementioned research background, this study employs a strategy that involves structural modification of both the carboxyl and amino terminals of ALA, in conjunction with the incorporation of the pharmacophore of iron chelator DFP. The objective is to design a novel class of ALA-HPO derivatives that synergistically exert antitumor effects by targeting both the mechanism of the haem biosynthesis pathway and the dysregulation of iron metabolism in tumour cells.

## Results and discussion

### Design of novel ALA-HPO derivatives

Preliminary studies indicate that esterification modifications at the carboxyl terminal of ALA enhance lipophilicity and membrane permeability, whereas amidation at the amino terminal improves chemical stability. Consequently, dual prodrugs that modify both terminals of ALA simultaneously are anticipated to exhibit superior drug-like properties and PDT activity. In terms of esterification, straight-chain alkyl esters show optimal performance, with PpIX yield increasing in proportion to the length of the alkyl chain, within a certain range^19^^,^^35^. Regarding amidation modifications, peptide derivatives conjugated with amino acids demonstrate enhanced efficacy; notably, only *L*-amino acid derivatives undergo hydrolysis by intracellular peptidases^36^^,^^37^. Among these, acidic or basic amino acids are less effective in producing PpIX, while neutral amino acids, particularly phenylalanine and leucine, yield higher levels of PpIX^20^^,^^38–40^. Furthermore, long-term research conducted by our group on HPO-derived iron chelators has revealed that the hydroxyl group at the 3-position and the ketone group at the 4-position are essential for iron chelation. Additionally, retaining the methyl group at the 2-position of DFP and coupling pharmacophores at the N1 position can augment both lipophilicity and iron chelation capability^28^^,^^41–45^.

**Figure 3.**
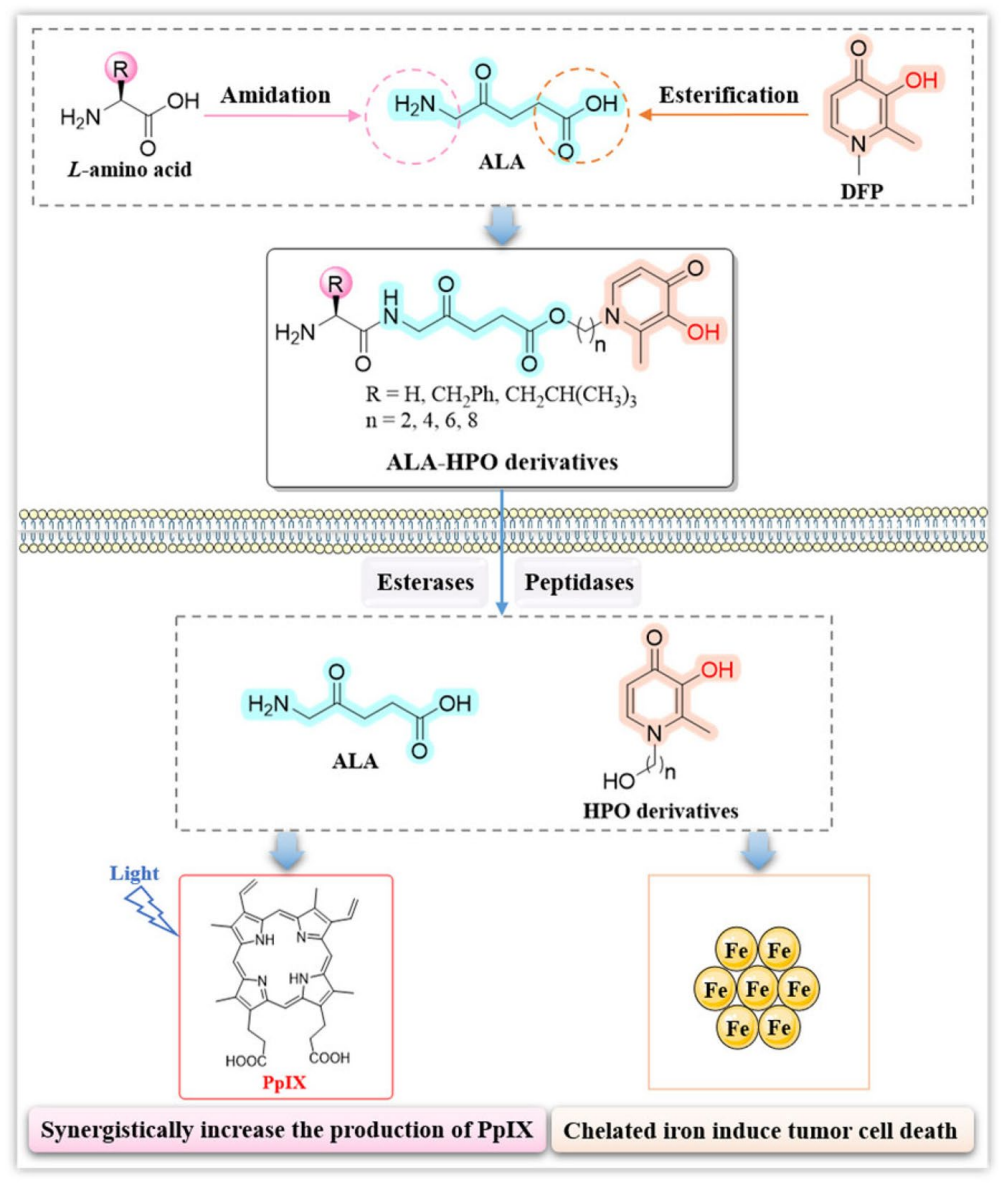
The design of novel ALA-HPO derivatives.

Therefore, based on previous studies, this work introduced a straight-chain alkyl ester at the N1 position of DFP – which is more favourable for enhancing activity – and linked it to the carboxyl terminal of ALA via an ester bond, while the amino group of ALA was conjugated to an *L*‑natural amino acid through an amide bond. Through modification of the chain length and type of amino acids, the physicochemical properties and photodynamic activity of the compounds can be improved. The ester and amide bonds can be hydrolysed by intracellular esterases and peptidases, respectively, resulting in the release of ALA and HPO-derived iron chelators that can work synergistically. Consequently, a novel class of dual prodrugs, ALA-HPO derivatives, with enhanced anti-tumour activity has been designed (Figure 3).

### Synthesis of ALA-HPO derivatives

**Figure 4.**
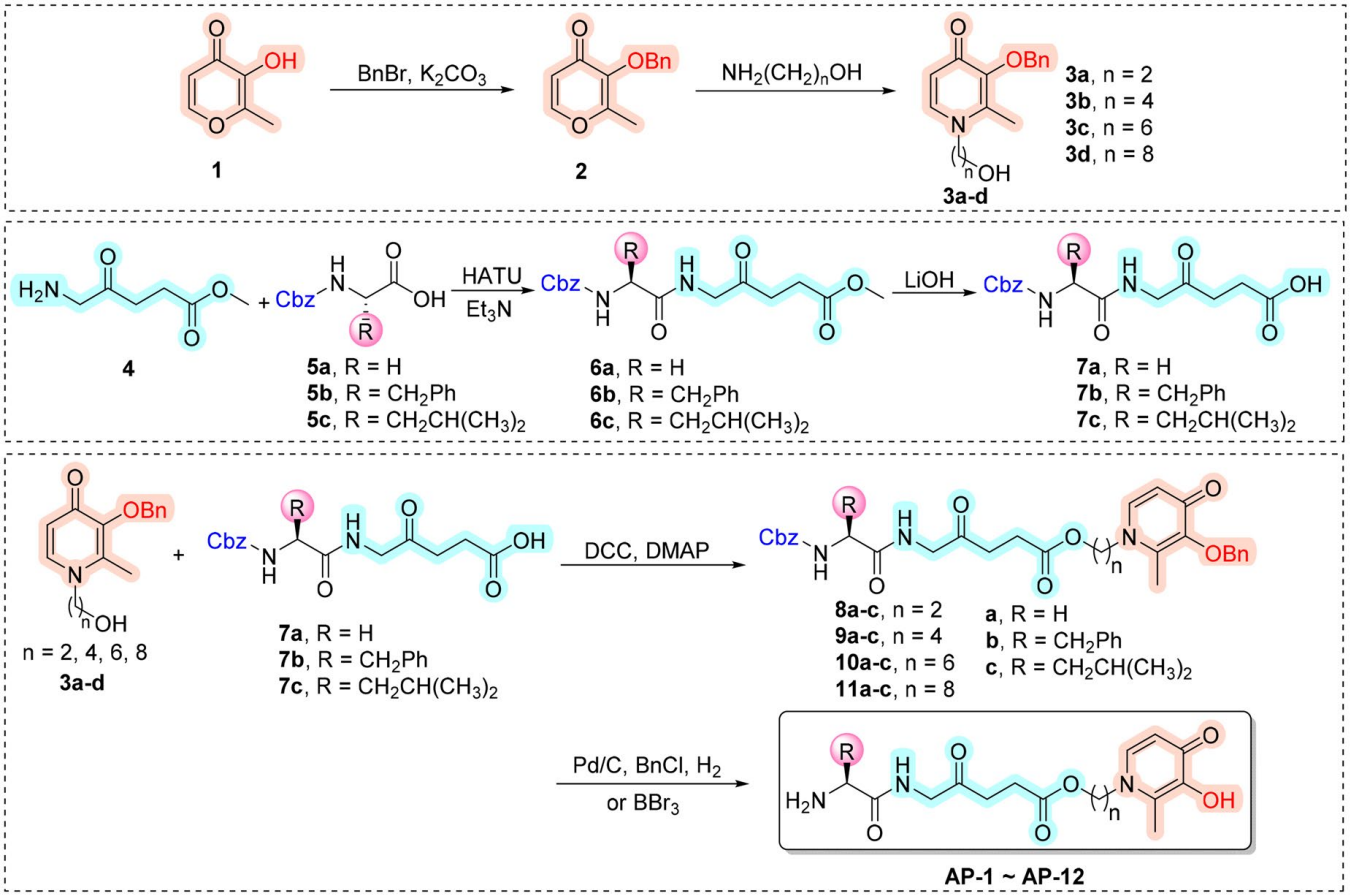
The synthetic strategies of ALA-HPO derivatives **AP-1–12**.

The synthesis route of compounds **AP-1–12**, depicted in Figure 4, encompasses three primary pathways: the synthesis of HPO intermediates, the synthesis of ALA intermediates, and the synthesis of the target compound.

Initially, maltol **1** is served as the starting material, and its hydroxyl group at the 3-position is protected using benzyl bromide to generate intermediate **2**. This intermediate is then reacted with linear alkylol amines of varying chain lengths under basic conditions, facilitated by sodium hydroxide, resulting in the formation of HPO intermediates **3a-d**.

In a separate pathway, methyl 5-aminolevulinic acid **4** is employed as the starting material, where *N,N,N’,N’*-tetramethyl-*O*-(7-azabenzotriazol-1-yl)uronium hexafluorophosphate (HATU) is used as the coupling agent and triethylamine acts as the acid scavenger. This combination enables an amide coupling reaction with *L*-glycine, *L*-phenylalanine, and *L*-leucine, which are protected with a benzyloxycarbonyl (Cbz) group, yielding intermediates **6a-c**. Finally, these intermediates undergo hydrolysis of the methyl ester using lithium hydroxide, producing ALA intermediates **7a-c**.

HPO intermediates **3a-d** and ALA intermediates **7a-c** are participated in a condensation reaction under dicyclohexylcarbodiimide (DCC) and dimethylaminopyridine (DMAP) conditions, yielding esters **8–11**. Subsequently, the benzyloxycarbonyl (Cbz) and benzyl groups are simultaneously removed using boron tribromide or Pd/C in the presence of H_2_, resulting in the production of compounds **AP-1–12**.

It is important to note that the final products **AP-1–12** resemble peptides and exhibit high polarity. Furthermore, the HPO-derived iron chelators show potent iron chelating capacity, leading to their tendency to chelate on thin-layer chromatography plates and silica gel columns. This characteristic renders conventional silica gel chromatography unsuitable for separation. Consequently, this study employed preparative high-performance liquid chromatography (HPLC) in conjunction with recrystallization for the separation and purification of the final product.

### Dark cytotoxicity assay

**Figure 5.**
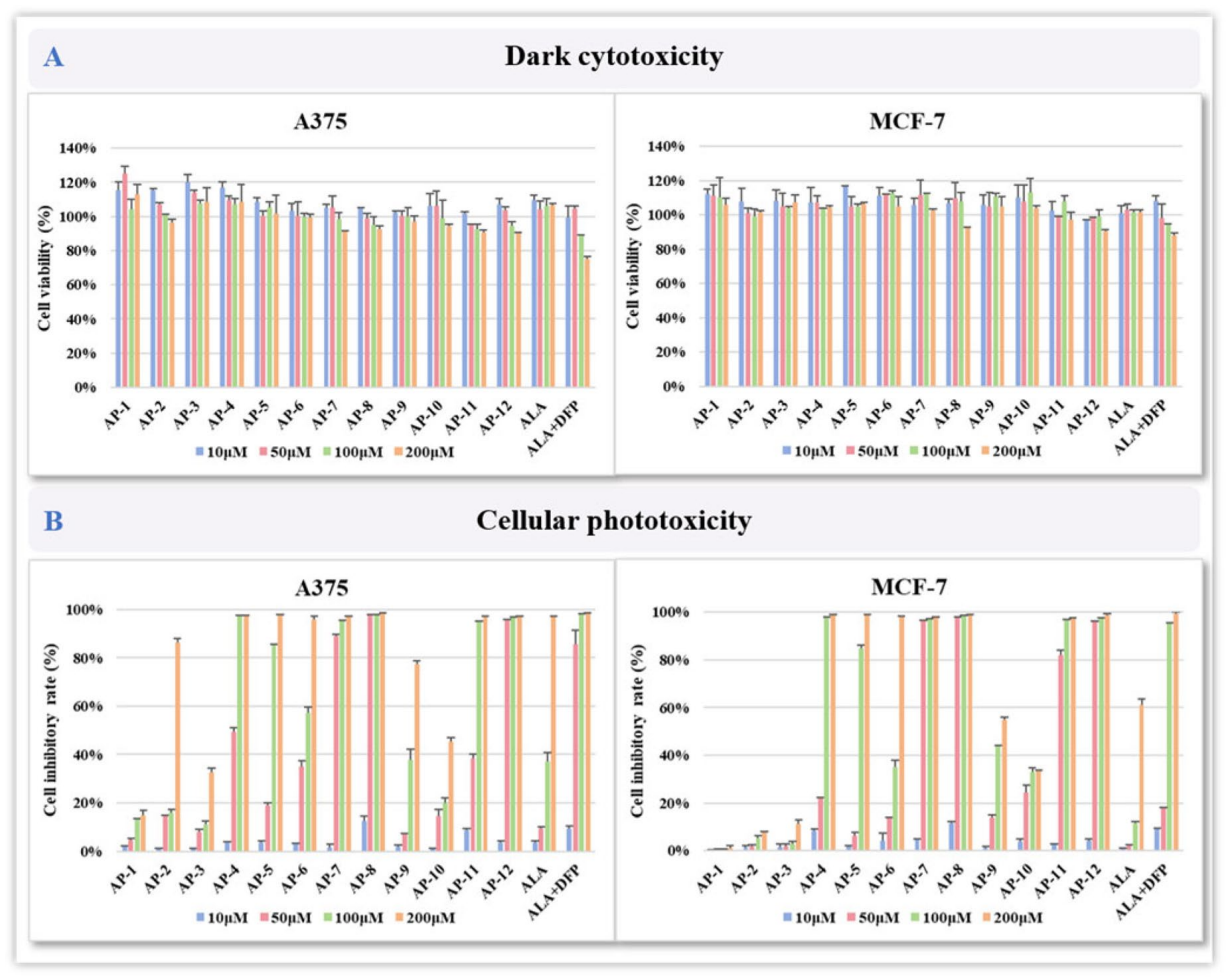
(A) The cell viability of compounds **AP-1–12**, ALA and ALA+DFP on A375 and MCF-7 cells at various concentrations in the dark for 4 h. (B) The cell inhibitory rate of compounds **AP-1–12**, ALA and ALA+DFP on A375 and MCF-7 cells at various concentrations for 4 h, followed by exposure to blue light (∼420 nm, light dose 5 J/cm^2^) for 5 min.

Cellular dark toxicity refers to cytotoxicity that occurs in the absence of light and is a critical factor in evaluating the efficacy of photosensitizers. Photosensitizers with negligible or low dark cytotoxicity allow normal cells and tissues to grow and remain unharmed in darkness, thereby ensuring their safety when not exposed to light. To assess this, the CCK-8 assay was utilised to determine the cell viability of the compounds **AP-1–12**, along with the positive controls ALA and ALA+DFP (molar ratio 1:1), across four concentrations (10 μM, 50 μM, 100 μM, and 200 μM) for the A375 and MCF-7 cell lines. According to existing literature and clinical studies, optimal phototherapy occurs approximately 4 h after ALA administration^46^^,^^47^ .Therefore, following treatment, cells were incubated with the compounds in the dark for 4 h before evaluating their viability (Figure 5A).

The results indicated that all target compounds **AP-1–12** and ALA did not exhibit significant dark cytotoxicity at the four concentrations tested in both cell lines, with cell viability exceeding 90%. However, the ALA+DFP combination group demonstrated slight dark cytotoxicity in the A375 cell line at 100 μM (cell viability of 89.02%) and pronounced dark cytotoxicity at 200 μM (cell viability of 75.04%). In the MCF-7 cell line, significant dark cytotoxicity was also observed at 200 μM, resulting in a cell viability of 88.03%. Consequently, it can be concluded that the safety profile of the ALA-HPO derivatives **AP-1–12** is markedly superior to that of the ALA+DFP combination therapy.

### Cellular phototoxicity assay

Phototoxicity refers to cell death induced by photosensitizers under specific light exposure, a key factor in evaluating the antitumor efficacy of photosensitizers. As mentioned in the introduction, ALA functions as a prodrug that, upon entering the cells, undergoes a multi-step metabolic conversion to the photosensitiser PpIX. PpIX has two main absorption peaks: the Soret band (∼410 nm, blue light) displays the maximum absorption peak but lower tissue penetration capability (micron level), while the Q band (∼630 nm, red light) has a weaker absorption peak but offers deeper tissue penetration (millimeter to centimetre level) ^48^. Research indicates that *in vitro* cell experiments frequently utilise blue light, as it maximises the excitation of PpIX, facilitating more efficient and rapid compound screening. In contrast, *in vivo* animal studies employ red light, offering superior tissue penetration. This approach minimises excessive damage to normal superficial tissues and more closely approximates clinical application conditions^49–51^.

Based on the above analysis, we conducted a preliminary screening and evaluation of the compounds **AP-1–12**, along with the positive drugs ALA and ALA+DFP combination, at four different concentrations (10 μM, 50 μM, 100 μM, and 200 μM). After a 4 h culture with A375 and MCF-7 cell lines, the cells were subjected to blue light irradiation (light dose of 5 J/cm^2^), and the cell proliferation inhibition was measured (Figure 5(B)). The results demonstrated that all compounds exhibited varying degrees of phototoxicity at different concentrations, with higher concentrations associated with increased phototoxicity. Analysis of the terminal amino acid substitutions revealed that phenylalanine substitutions (**AP-5–8**) were more effective than leucine substitutions (**AP-9–12**), which, in turn, were more effective than glycine substitutions (**AP-1–4**). Additionally, a trend was observed indicating that longer chain lengths generally resulted in greater phototoxicity. Importantly, the phototoxicity of the 8 compounds (**AP-4–9**, and **AP-11–12**) was significantly greater than that of ALA, while the phototoxicity of 4 compounds (**AP-7–8**, and **AP-11-AP-12**) was more significant than that of the ALA+DFP combination.

Subsequently, we conducted tests with different concentration gradients for each compound to determine their inhibition rates, which yielded the IC_50_ values presented in Table 1. Consistent with the trends observed during preliminary screening, the IC_50_ values of the 8 compounds (**AP-4–9**, and **AP-11–12**) were all lower than that of ALA. This finding suggests that most ALA-HPO hybrids exhibit superior efficacy compared to ALA alone. Notably, 4 compounds (**AP-7–8**, **AP-11–12**) displayed IC_50_ values significantly lower than that of the ALA+DFP combination, indicating that ALA-HPO hybrids can offer improved outcomes compared to the ALA+DFP combination. The compounds **AP-8** (A375: IC_50_ = 25.55 ± 0.82 μM; MCF-7: IC_50_ = 28.38 ± 0.21 μM), and **AP-12** (A375: IC_50_ = 26.34 ± 0.06 μM; MCF-7: IC_50_ = 32.47 ± 0.26 μM) emerged as particularly effective candidates. It is hypothesised that both compounds exhibit strong lipophilicity, which enhances their cellular absorption and facilitates the release of the PpIX precursor (ALA) and the iron chelator HPO, thereby leading to the production of high concentrations of PpIX.

**Table 1.** IC_50_ values of compounds **AP-1–12**, ALA and ALA+DFP.

Compound	IC_50_ ± *SD* (μM, *n* = 3)	
	A375	MCF-7
**AP-1**	564.87 ± 27.68	975.53 ± 30.94
**AP-2**	141.83 ± 7.25	282.07 ± 13.98
**AP-3**	230.37 ± 4.83	364.40 ± 29.45
**AP-4**	51.13 ± 1.49	56.91 ± 0.28
**AP-5**	88.75 ± 1.98	76.88 ± 0.99
**AP-6**	80.58 ± 1.17	109.47 ± 1.18
**AP-7**	39.74 ± 0.79	36.18 ± 0.42
**AP-8**	25.55 ± 0.82	28.38 ± 0.21
**AP-9**	124.63 ± 6.37	142.23 ± 1.33
**AP-10**	208.20 ± 6.62	221.00 ± 34.22
**AP-11**	53.88 ± 0.15	44.58 ± 2.15
**AP-12**	26.34 ± 0.06	32.47 ± 0.26
ALA	141.80 ± 8.19	194.00 ± 6.08
ALA+DFP	44.23 ± 0.68	82.69 ± 0.38

### Drug-likeness assessment

To further assess the relationship between the drug-like properties, lipophilicity, and phototoxicity of the target compounds, we utilised the chemoinformatics software Molinspiration (http://www.molinspiration.com) to predict and analyse the molecular characteristics of these compounds. Previous studies indicate that the miLog P (lipophilicity) values calculated with this software for HPO-type compounds are more closely aligned with experimental values compared to those obtained from other software illustrated in Table 2^45^.

**Table 2.** The drug-likeness evaluation of all target compounds.

Compound	Molecular weight	miLog *P*	TPSA	HBD	HBA
**AP-1**	339.35	−2.92	140.73	4	9
**AP-2**	367.40	−2.38	140.73	4	9
**AP-3**	395.46	−1.37	140.73	4	9
**AP-4**	423.51	−0.36	140.73	4	9
**AP-5**	429.47	−1.56	140.73	4	9
**AP-6**	457.53	−1.02	140.73	4	9
**AP-7**	485.58	−0.01	140.73	4	9
**AP-8**	513.63	1.00	140.73	4	9
**AP-9**	395.46	−1.71	140.73	4	9
**AP-10**	423.51	−1.17	140.73	4	9
**AP-11**	451.56	−0.16	140.73	4	9
**AP-12**	479.62	0.85	140.73	4	9
ALA	117.10	−1.17	80.39	3	4
DFP	139.15	−0.60	42.23	1	3

All compounds demonstrate favourable drug-like properties. The miLog *p* values reveal that compounds substituted with phenylalanine (**AP-5–8**) possess greater lipophilicity than those substituted with leucine (**AP-9–12**), which are themselves more lipophilic than those substituted with glycine (**AP-1–4**). Within groups of compounds having the same amino acid substitution, increased chain length correlates with enhanced lipophilicity, with compound **AP-8** exhibiting the highest lipophilicity, in agreement with cytotoxicity assay results. Moreover, the topological polar surface area (TPSA) quantifies the contribution of polar fragments to the molecular surface, which in turn reflects the non-specific toxicity and bioavailability of the compounds. A TPSA < 75 Å^2^ is associated with an elevated risk of non-specific toxicity, indicating that the target compounds generally exhibit low toxicity^52^^,^^53^. Additionally, for compounds within the molecular weight range of 255–580, a TPSA < 154 Å^2^ and a Log *P* > 0 predict intestinal absorption in humans^54^ .Notably, only compounds **AP-8** and **AP-12** demonstrate significant potential for intestinal absorption. Consequently, potent lipophilicity and favourable absorption are critical factors that enhance the PDT activity of ALA-HPO compounds.

It is important to note that the drug-likeness predictions aim to demonstrate the theoretical lipophilicity and intestinal absorption potential of the compounds **AP-8** and **AP-12**. This suggests that these compounds possess favourable tissue permeability and oral bioavailability potential. Lipophilicity and permeability of compound are critical factors in enhancing the efficacy of PDT. However, in the subsequent evaluation using animal models, we opted for intratumoral injection rather than oral administration to minimise pharmacokinetic variables and concentrate on validating the local efficacy.

### PpIX fluorescence kinetics assay

The quantity of PpIX generated following the cellular absorption and metabolism of ALA derivatives directly affects the intensity of phototoxicity and serves as a crucial parameter for assessing the effectiveness of PDT. Consequently, we initially evaluated the fluorescence intensity of PpIX after administering two selected compounds (**AP-8** and **AP-12**) as well as positive controls (ALA and ALA+DFP) at four different concentrations (10 μM, 50 μM, 100 μM, and 200 μM) with the A375 and MCF-7 cell lines over a 4-h incubation period, which indicates the PpIX production levels. Subsequently, based on these initial results, we selected a concentration of 50 μM to investigate the variations in PpIX fluorescence intensity over multiple incubation durations (2 h, 4 h, 6 h, 8 h, 12 h, and 24 h) for the compounds **AP-8**, **AP-12**, ALA and ALA+DFP with both A375 and MCF-7 cell lines.

**Figure 6.**
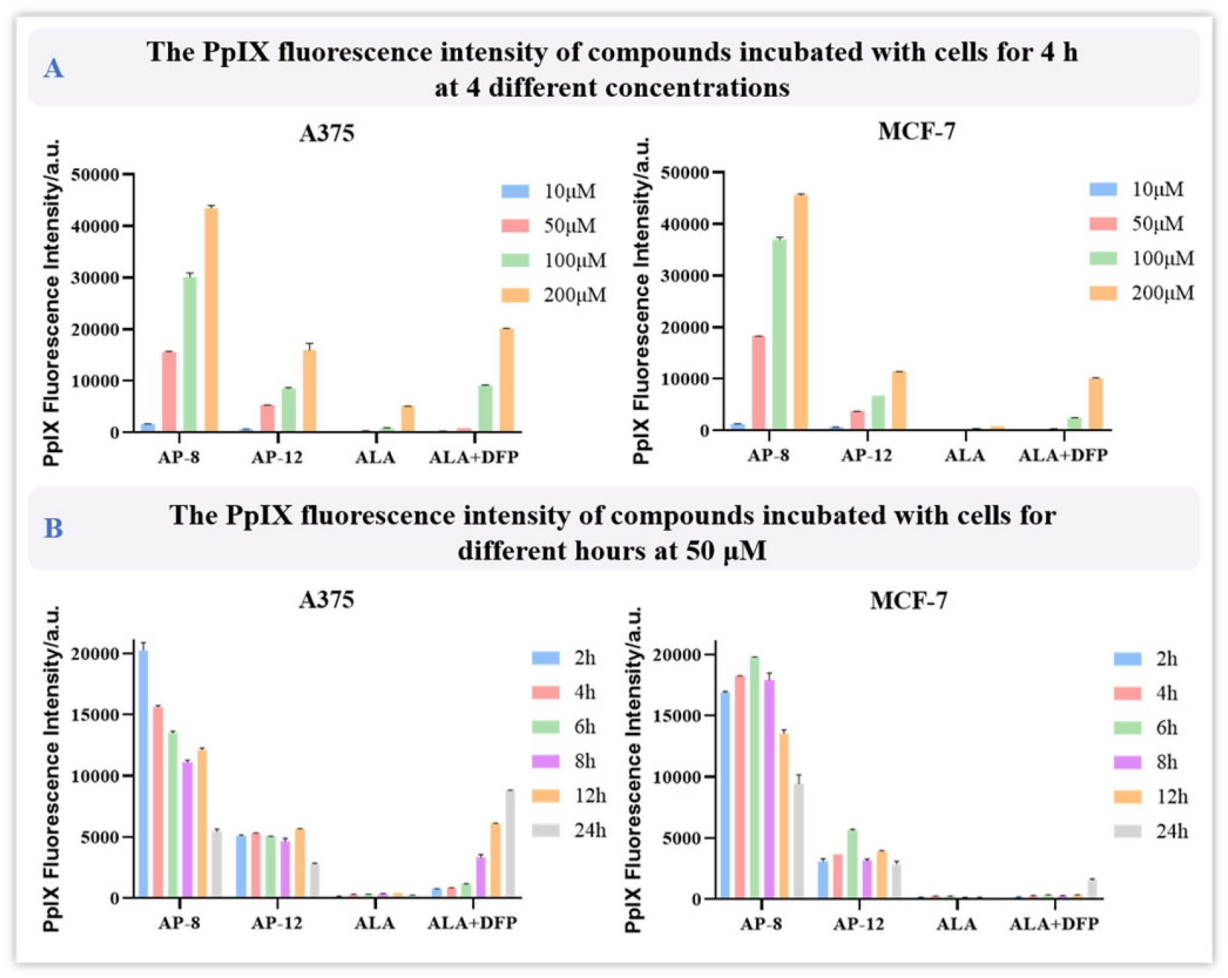
(A) The PpIX fluorescence intensity of compounds **AP-8**, **AP-12**, ALA and ALA+DFP incubated with A375 and MCF-7 cells at various concentrations in the dark for 4 h. (B) The PpIX fluorescence intensity of compounds **AP-8**, **AP-12**, ALA and ALA+DFP incubated with A375 and MCF-7 cells at 50 μM in the dark for various time intervals.

The results are presented in Figure 6. After a 4-h incubation, the levels of PpIX produced by the compounds **AP-8** and **AP-12**, as well as the positive controls (ALA and ALA+DFP), displayed a dose-dependent relationship, with fluorescence intensity increasing progressively with higher concentrations. Notably, compound **AP-12** exhibited slightly superior performance compared to the positive control ALA+DFP and demonstrated a significant advantage over ALA. The optimised compound **AP-8** showed a markedly higher yield of PpIX, significantly exceeding the outputs of both **AP-12** and the positive controls. Across the four concentrations tested (10 μM, 50 μM, 100 μM, and 200 μM), the fluorescence intensities recorded for the A375 cell line were 19.00, 52.49, 34.55, and 8.58 times greater than those of ALA, and 6.67, 18.16, 3.31, and 2.16 times greater than those of ALA+DFP, respectively. In the MCF-7 cell line, the fluorescence intensities were recorded as 9.41, 69.75, 109.19, and 60.02 times that of ALA, and 9.25, 65.57, 15.16, and 4.49 times that of ALA+DFP, respectively.

At a concentration of 50 μM, the production of PpIX by **AP-8** and **AP-12**, along with the positive controls (ALA and ALA+DFP), demonstrated a time-dependent relationship following cell incubation. For the positive controls ALA and ALA+DFP, fluorescence intensity showed a gradual increase with prolonged incubation, although this trend was not particularly pronounced. Compound **AP-12** exhibited slightly enhanced activity compared to the positive controls. In the A375 cell line, fluorescence intensity remained relatively stable between 2 and 12 h but exhibited a decline at 24 h, likely due to increased dark cytotoxicity associated with prolonged incubation, leading to cell death. Conversely, in the MCF-7 cell line, fluorescence intensity gradually increased from 2 to 6 h, before decreasing as a result of enhanced dark cytotoxicity during extended incubation.

In stark contrast, the optimised compound **AP-8** yielded significantly higher levels of PpIX across all incubation times, far surpassing the results of **AP-12** and the positive controls. In the A375 cell line, an exceptionally high fluorescence intensity was recorded after just 2 h of incubation, which was 3.96 times greater than that of **AP-12**, 145.72 times greater than ALA, and 26.53 times greater than ALA+DFP. Despite a decrease in fluorescence intensity due to increased dark cytotoxicity with longer incubation, values remained considerably higher than those of **AP-12** and the positive controls within the 12-h duration. Similarly, in the MCF-7 cell line, fluorescence intensity steadily increased from 2 to 6 h; at the 6-h mark, intensity was 3.50 times that of **AP-12**, 85.14 times that of ALA, and 58.21 times that of ALA+DFP. Following this period, further extended incubation again resulted in reduced fluorescence intensity due to increased dark toxicity, yet levels continued to be significantly greater than those of **AP-12** and the positive controls. Based on these findings, **AP-8** was selected as the optimised compound for subsequent *in vivo* photodynamic anti-tumour experiments.

### Structure-activity relationship analysis

**Figure 7.**
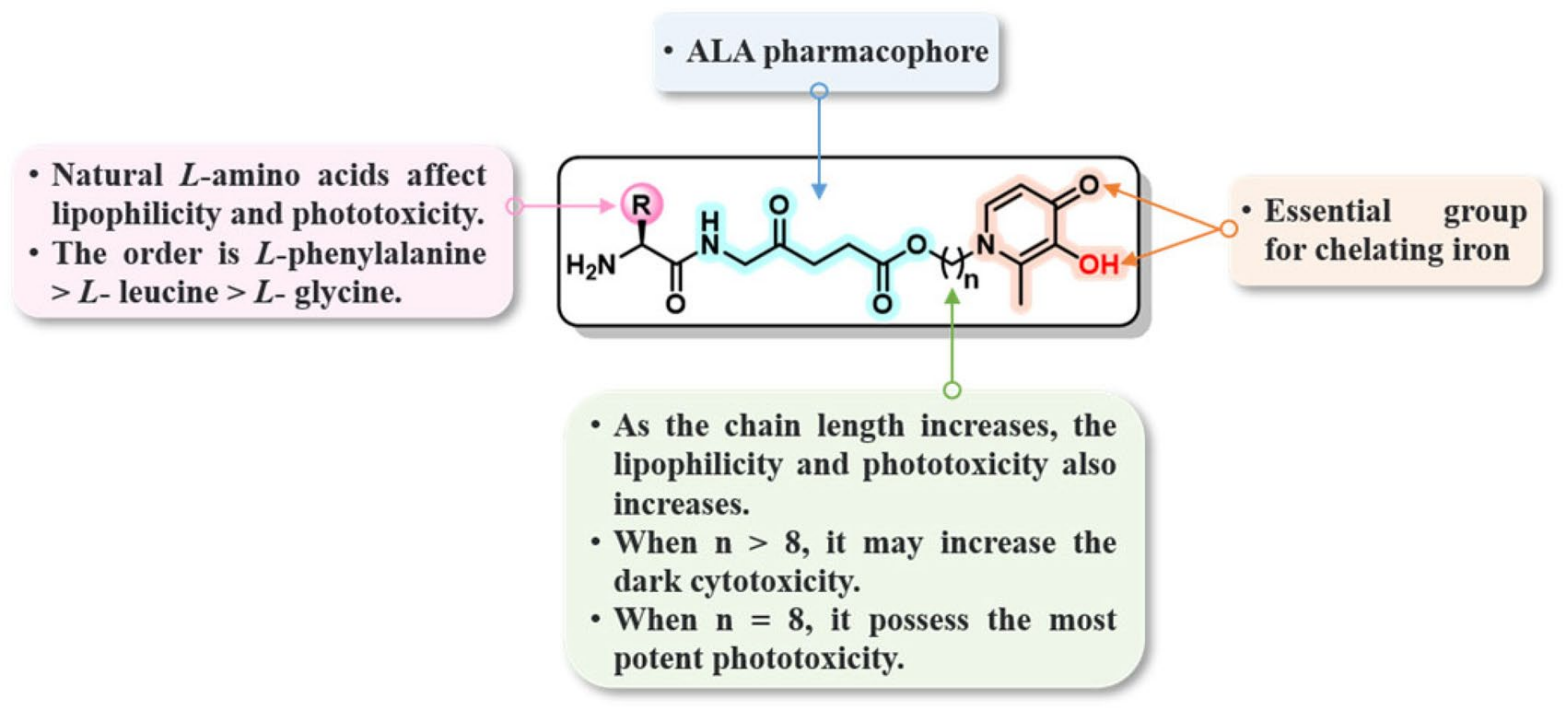
The structure-activity relationships of ALA-HPO derivatives.

Based on the *in vitro* experimental results, the structure-activity relationships of ALA-HPO derivatives are summarised in Figure 7.

1. The presence of 3-hydroxy and 4-keto groups on the pyridone is essential for iron chelation.
2. At the N1 position of the pyridone, increasing the chain length enhances lipophilicity and dark cytotoxicity. At *n* = 8 and a concentration of 200 μM, compounds substituted with *L*-phenylalanine and *L*-leucine exhibit slight dark cytotoxicity, yet cell viability remains above 90%, indicating minimal cellular damage; this dark cytotoxicity is lower than that observed with ALA+DFP at the same concentration. However, for derivatives with *n* > 8, an increase in dark cytotoxicity may occur. Consequently, synthesising compounds with longer chain lengths is unnecessary, as such lengths also complicate the synthesis, separation, and purification processes.
3. At the N1 position of the pyridone, phototoxicity also increases with chain length, peaking at n = 8, which significantly exceeds the phototoxicity of both ALA and ALA+DFP.
4. The substitution of terminal natural *L*-amino acids impacts both lipophilicity and phototoxicity, with *L*-phenylalanine showing superior effects compared to *L*-leucine, which in turn is more effective than *L*-glycine. Therefore, ALA-HPO derivatives, which are linked *via* ester bonds to ALA and HPO, and *via* amide bonds to natural *L*-amino acids, enable the cleavage of ester and amide bonds within cells. This mechanism facilitates the release of ALA and HPO, resulting in synergistic enhancement of PpIX accumulation and anti-tumour activity, thereby outperforming the combination therapy of ALA+DFP.

### *In vivo* photodynamic anti-tumour assay

A subcutaneous melanoma model in BALB/c nude mice, established with A375 cells, was employed to further investigate the *in vivo* photodynamic anti-tumour effects of **AP-8**. When the tumour volume reached between 50 and 100 mm³, the mice were randomly divided into six groups: control group, control + light group, ALA group, ALA+light group, **AP-8** group and **AP-8**+light group. Both ALA and **AP-8** were administered via intratumoral injection at a dose of 15 mg/kg once a day, one hour before red light exposure (635 nm, light dose of 180 J/cm^2^) for 7 consecutive days. Mouse body weight and tumour volume were recorded daily. On the seventh day, after euthanizing the mice, the tumours were excised, weighed, measured for volume, photographed, and tissue samples were collected for haematoxylin and eosin (H&E) staining analysis.

**Figure 8.**
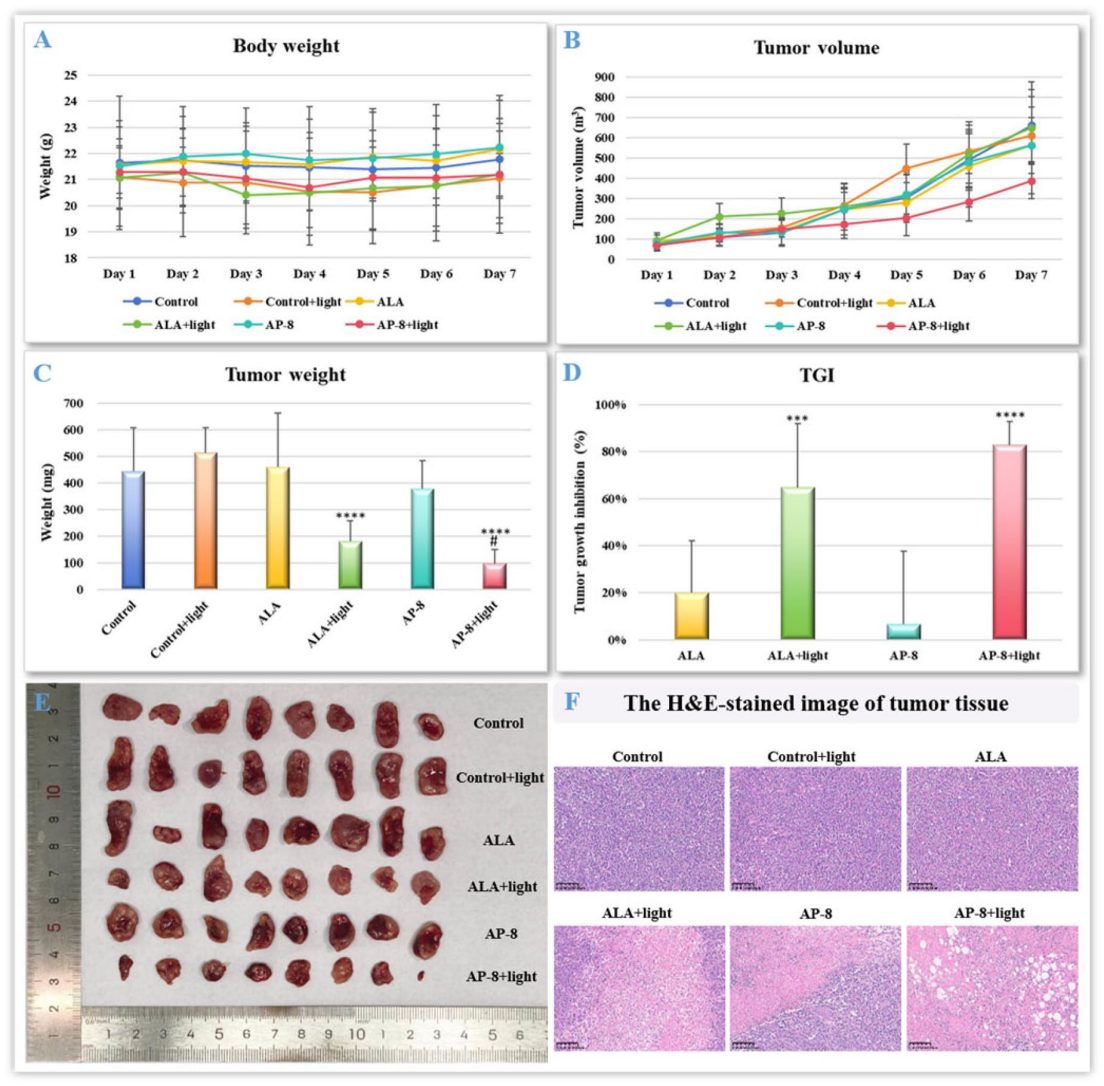
(A) The body weight of mice during treatment; (B) The tumour volume of mice during treatment; (C) The isolated tumour weight of mice on the last day of the experiment (****p < 0.0001, Control + light group *vs*. Treatment groups; ^#^*p* < 0.05, ALA+light group *vs*. AP-8 + light group); (D) The tumour growth inhibition (TGI) of the experimental groups (***p < 0.001, ****p < 0.0001, Control + light group *vs*. Treatment groups); (E) The photos of tumours; (F) The H&E-stained images of tumour tissues.

The average body weight of the mice consistently ranged from approximately 20–22 g per day, without significant fluctuations, thereby indicating stable experimental conditions and the biosafety of the protocol. Additionally, tumour growth did not result in other malignant lesions (Figure 8(A)). The tumour volume in both the control group and the non-irradiated groups exhibited a rapid increase over time, suggesting that neither light exposure nor medication alone effectively exerted antitumor effects. In contrast, the increase in tumour volume was significantly slowed in both the ALA+light group and the **AP-8**+light group, demonstrating their ability to inhibit tumour growth, with **AP-8** showing superior efficacy (Figure 8(B)).

Analysis of tumour weight and volume data at the experimental endpoint indicated that the control group (443.88 ± 164.43 mg) and the control + light group (513.50 ± 94.42 mg) exhibited the heaviest tumours. Moreover, the ALA group (459.25 ± 204.35 mg, TGI = 20.05% ± 22.09%) and the **AP-8** group (378.50 ± 105.04 mg, TGI = 6.74% ± 30.74%) also presented relatively heavy tumour weight and exhibited mild inhibitory effects on tumour growth, with **AP-8** demonstrating a preferable safety profile. In contrast, the ALA+light group (181.00 ± 76.43 mg, TGI = 64.74% ± 27.18%) and the AP-8 + light group (98.13 ± 51.96 mg, TGI = 82.74% ± 10.14%) revealed a significant reduction in tumour weight. Notably, the **AP-8**+light group displayed the lightest tumours and the most pronounced capability to inhibit tumour growth (Figure 8(C,E)).

Histological analysis demonstrated that the control group and the non-irradiated groups exhibited a higher density of tumour cells, characterised by larger nuclei and active cell division (Figure 8(F)). In contrast, the ALA+light group and the **AP-8**+light group displayed a decreased density of tumour cells, accompanied by signs of necrosis, such as cell degradation, loss of cell membrane integrity, and the presence of residual cellular debris. Notably, the extent of necrosis in tumour cells was more pronounced in the **AP-8**+light group.

## Conclusion

ALA-based PDT has extensive applications for the treatment of superficial tumours. Enhancing the drug-like properties of ALA while increasing PpIX yield is vital for improving its photodynamic anti-tumour efficacy. Iron chelators have been demonstrated to synergistically enhance PpIX production by inhibiting ferrochelatase, when used alongside ALA and can also induce tumour cell death by chelating intracellular iron. This combination strategy has been validated by multiple studies.

In this study, a series of novel ALA-HPO derivatives was designed and synthesised using rational drug design and pharmacophore fusion principles. The conjugates feature ALA linked to the 1‑position of HPO through an ester bond, with varying alkyl chain lengths and amino acid substituents introduced to fine‑tune stability and lipophilicity. Most of the compounds exhibited greater activity than ALA, with half showing enhanced efficacy compared to the combination of ALA and DFP. Among them, compound **AP-8**, featuring an 8-carbon chain and substituted with *L*-phenylalanine, demonstrated the most potent phototoxicity and highest PpIX levels, achieving approximately 145‑fold higher PpIX levels than ALA alone. PpIX fluorescence kinetics assay confirmed that **AP‑8** is effectively taken up by tumour cells and hydrolysed to release both ALA and HPO, leading to cooperative PpIX upregulation. Furthermore, the light‑dependent cytotoxicity indirectly supports that the accumulated PpIX generates sufficient singlet oxygen and ROS upon irradiation, thereby killing tumour cells. This mechanism is highly consistent with the iron chelation synergistic enhancement pathway reported in the literature.

Moreover, *in vivo* anti‑tumour assays demonstrated that **AP‑8** achieved a potent TGI rate of 84.74% without significant body weight loss, indicating a favourable therapeutic window. Collectively, our findings validate the ALA‑HPO prodrug strategy as an effective approach for co‑delivering ALA and an iron chelator, and identify **AP‑8** as a promising lead candidate for further development. Future studies will focus on comprehensive pharmacokinetic profiling and tissue distribution to fully assess its translational potential.

## Experimental methods

### Chemistry

All chemicals and solvents were purchased from Aladdin, Energy, Bidepharm, Macklin or Sigma Aldrich and were utilised without any subsequent purification. Silica gel (100–200, 200–300 mesh, Qingdao Haiyang Chemical Co., Ltd., China) was utilised for column chromatography. Precoated silica gel GF254 plates (Qingdao Haiyang Chemical Co., Ltd., China) were employed for thin-layer chromatography (TLC). The progress of the reaction was monitored using TLC, and visualisation was achieved with UV light or iodine staining. Melting points were determined with an OptiMelt MPA100 digital melting point apparatus.^1^H NMR and ^13^C NMR spectra were recorded on Bruker instruments (500 MHz and 125 MHz, respectively), with tetramethylsilane used as the internal standard. High-resolution mass spectra were obtained using an Agilent 1290LC-6530QTOF instrument, employing electrospray ionisation as the ion source. The purity of all compounds was evaluated through NMR spectroscopy and HRMS analysis. Compounds **AP-1–12** were purified by preparative HPLC (Waters 2535Q, UV detection at λ = 264 nm), utilising a Waters SunFire Prep C18 OBD column (19 × 250 mm, 10 μm) and eluting at a flow rate of 5 ml/min with acetonitrile/water (25:75, V/V) as mobile phase. All target compounds evaluated in biological assays possessed a purity of ≥ 95% as determined by HPLC.

#### General procedure for the preparation of HPO intermediates 3a-d

To a 250 ml round-bottom flask was added maltol **1** (60 mmol), benzyl bromide (180 mmol), anhydrous K_2_CO_3_ (180 mmol) and acetone (100 ml) sequentially. The resulting mixture was refluxed for 8 h and monitored by TLC. After the reaction was completed, the mixture was subjected to vacuum concentration to obtain the solid. The solid was dissolved in water (100 ml) and then extracted with DCM (100 ml × 4). The combined organic layers were dried over Na_2_SO_4_ and concentrated under reduced pressure. The resulting residue was further purified using thin layer chromatography (PE:EA = 4:1–2:1) to obtain yellow oil **2**, yield 97%.^1^H NMR (400 MHz, CDCl_3_) δ 7.59 (d, *J* = 6.0 Hz, 1H), 7.42–7.36 (m, 2H), 7.36–7.31 (m, 3H), 6.37 (d, *J* = 5.6 Hz, 1H), 5.16 (s, 2H), 2.09 (s, 3H).

To a 100 ml round-bottom flask was added intermediate **2** (10 mmol), alkylol amine with different chain lengths (25 mmol), EtOH (15 ml), H_2_O (15 ml) and 2 M NaOH (1 ml) sequentially. The resulting mixture was refluxed for 18–20 h and monitored by TLC. After the reaction was completed, the mixture was subjected to vacuum concentration to obtain the solid. The solid was dissolved in water and then the pH was adjusted to 1 with concentrated HCl. The solution was washed with ether (25 ml × 2), the aqueous layer was adjusted to pH 9 with 10 M NaOH, and then extracted multiple times with DCM (50 ml). The combined organic layers were dried over Na_2_SO_4_ and concentrated under reduced pressure. The resulting residue was recrystallized with EA to obtain pale yellow solids **3a-d**.

**3a**, pale yellow solid, yield 69%; m.p. 148–150 °C. ^1^H NMR (500 MHz, CDCl_3_) δ 7.39–7.34 (m, 2H), 7.33 (d, *J* = 2.5 Hz, 1H), 7.32–7.29 (m, 3H), 6.17 (d, *J* = 7.5 Hz, 1H), 4.95 (s, 2H), 3.84 (t, *J* = 4.5 Hz, 4H), 2.11 (s, 3H).

**3b**, pale yellow solid, yield 71%; m.p. 104–106 °C. ^1^H NMR (500 MHz, CDCl_3_) δ 7.41–7.34 (m, 2H), 7.33–7.28 (m, 3H), 7.23 (d, *J* = 7.5 Hz, 1H), 6.33 (d, *J* = 7.5 Hz, 1H), 5.14 (s, 2H), 3.79 (q, *J* = 8.5 Hz, 2H), 3.65 (q, *J* = 6.0 Hz, 2H), 2.06 (s, 3H), 1.76–1.68 (m, 2H), 1.54–1.49 (m, 2H).

**3c**, pale yellow solid, yield 79%; m.p. 91–93 °C. ^1^H NMR (500 MHz, CDCl_3_) δ 7.37 (m, 2H), 7.28 (m, 3H), 7.16 (d, *J* = 7.5 Hz, 1H), 6.38 (d, *J* = 7.5 Hz, 1H), 5.18 (s, 2H), 3.69 (t, *J* = 6.5 Hz, 2H), 3.61 (t, *J* = 6.0 Hz, 2H), 2.04 (s, 3H), 1.65–1.57 (m, 2H), 1.56–1.50 (m, 2H), 1.40–1.34 (m, 2H), 1.30–1.26 (m, 2H).

**3d**, pale yellow solid, yield 73%; m.p. 90–92 °C. ^1^H NMR (500 MHz, CDCl_3_) δ 7.41–7.37 (m, 2H), 7.33–7.27 (m, 3H), 7.16 (d, *J* = 7.5 Hz, 1H), 6.41 (d, *J* = 7.5 Hz, 1H), 5.21 (s, 2H), 3.70 (t, *J* = 7.5 Hz, 2H), 3.63 (t, *J* = 6.5 Hz, 2H), 2.06 (s, 3H), 1.64–1.58 (m, 2H), 1.56–1.52 (m, 2H), 1.35–1.31 (m, 4H), 1.30–1.24 (m, 4H).

#### General procedure for the preparation of ALA intermediates 7a-c

To a 100 ml round-bottom flask was added 5-aminolevulinic acid methyl ester **4** (20 mmol), Cbz-protected *L*-amino acid **5a-c** (24 mmol), HATU (24 mmol) and DMF (60 ml) sequentially. The resulting mixture was stirred at −5 °C for 30 min and then slowly dropwise added triethylamine (60 mmol). Upon completion of the addition, the mixture was stirred at room temperature overnight and monitored by TLC. After the reaction was completed, the mixture was filtered and the filtrate was subjected to vacuum concentration to obtain the solid. The solid is dissolved in water and then extracted with DCM (50 ml × 4). The combined organic layers were washed with dilute HCl, saturated aqueous NaHCO_3_ and water, dried over Na_2_SO_4_ and concentrated under reduced pressure to obtain pale yellow solids **6a-c**.

**6a**, pale yellow solid, yield 84%; m.p. 103–105 °C. ^1^H NMR (500 MHz, CDCl_3_) δ 7.37–7.28 (m, 5H), 6.84 (s, 1H), 5.61 (s, 1H), 5.11 (s, 2H), 4.18 (d, *J* = 4.0 Hz, 2H), 3.90 (d, *J* = 4.2 Hz, 2H), 3.65 (s, 3H), 2.71 (t, *J* = 6.5 Hz, 2H), 2.63 (t, *J* = 6.0 Hz, 2H).

**6b**, pale yellow solid, yield 78%; m.p. 105–107 °C. ^1^H NMR (500 MHz, CDCl_3_) δ 7.37–7.30 (m, 5H), 7.28 (d, *J* = 7.0 Hz, 2H), 7.24–7.21 (m, 1H), 7.17 (d, *J* = 7.0 Hz, 2H), 6.54 (s, 1H), 5.30 (s, 1H), 5.07 (d, *J* = 3.0 Hz, 2H), 4.47 (s, 1H), 4.22–4.03 (m, 2H), 3.67 (s, 3H), 3.09 (d, *J* = 6.5 Hz, 2H), 2.70–2.66 (m, 2H), 2.65–2.60 (m, 2H).

**6c**, pale yellow solid, yield 80%; m.p. 120–122 °C. ^1^H NMR (500 MHz, CDCl_3_) δ 7.32–7.27 (m, 5H), 5.41 (d, *J* = 8.0 Hz, 1H), 5.14–5.02 (m, 2H), 4.30–4.22 (m, 1H), 4.15 (d, *J* = 4.5 Hz, 2H), 3.65 (s, 3H), 2.70 (t, *J* = 6.0 Hz, 2H), 2.62 (t, *J* = 6.0 Hz, 2H), 1.71–1.60 (m, 2H), 1.57–1.47 (m, 1H), 0.92 (s, 3H), 0.91 (s, 3H).

To a 100 ml round-bottom flask was added intermediates **6a-c** (2 mmol), MeOH (4 ml), H_2_O (4 ml) and LiOH (2.2 mmol) sequentially. The resulting mixture was stirred at room temperature for 20–30 min and monitored by TLC. After the reaction was completed, the mixture was adjusted to pH 2 with concentrated HCl, resulting in the precipitation of white solids. The solids were then filtered, washed with water, and dried under vacuum, yielding white solids **7a-c**.

**7a**, white solid, yield 77%; m.p. 158–159 °C. ^1^H NMR (500 MHz, DMSO-*d*_6_) δ 12.18 (s, 1H), 8.13 (t, *J* = 5.5 Hz, 1H), 7.51 (t, *J* = 6.0 Hz, 1H), 7.39–7.28 (m, 5H), 5.03 (s, 2H), 3.96 (d, *J* = 5.5 Hz, 2H), 3.66 (d, *J* = 6.5 Hz, 2H), 2.64 (t, *J* = 6.5 Hz, 2H), 2.41 (t, *J* = 6.5 Hz, 2H).

**7b**, white solid, yield 73%; m.p. 74–76 °C. ^1^H NMR (500 MHz, DMSO-*d*_6_) δ 12.20 (s, 1H), 8.39 (t, *J* = 5.0 Hz, 1H), 7.57 (d, *J* = 8.5 Hz, 1H), 7.35–7.26 (m, 5H), 7.27–7.19 (m, 5H), 4.94 (s, 2H), 4.30 (td, *J* = 10.5, 4.0 Hz, 1H), 4.04–3.90 (m, 2H), 3.04 (dd, *J* = 13.5, 3.5 Hz, 1H), 2.76 (dd, *J* = 13.5, 11.0 Hz, 1H), 2.63 (t, *J* = 6.5 Hz, 2H), 2.41 (t, *J* = 6.5 Hz, 2H).

**7c**, white solid, yield 71%; m.p. 83–84 °C. ^1^H NMR (500 MHz, DMSO-*d*_6_) δ 12.17 (s, 1H), 8.18 (t, *J* = 5.5 Hz, 1H), 7.45 (d, *J* = 8.0 Hz, 1H), 7.35–7.30 (m, 5H), 5.02 (d, *J* = 2.5 Hz, 2H), 4.07 (q, *J* = 9.0 Hz, 1H), 3.92 (qd, *J* = 18.0, 5.5 Hz, 2H), 2.62 (d, *J* = 7.0 Hz, 2H), 2.40 (t, *J* = 6.5 Hz, 2H), 1.66–1.60 (m, 1H), 1.49–1.41 (m, 2H), 0.87 (d, *J* = 6.5 Hz, 3H), 0.85 (d, *J* = 6.5 Hz, 3H).

#### General procedure for the preparation of ALA-HPO intermediates 8–11

To a 100 ml round-bottom flask was added ALA intermediates **7a-c** (1.1 mmol), anhydrous DCM (100 ml), anhydrous DMF (2.5 ml), DCC (1 mmol) and DMAP (0.2 mmol) sequentially in the atmosphere of N_2_. The resulting mixture was stirred at room temperature for 50 min and then the dry DCM solution of the intermediate **3a-d** (1 mmol) was added dropwise through a pressure-equalizing dropping funnel. The resulting mixture was stirred at room temperature overnight and monitored by TLC. After the reaction was completed, the mixture was subjected to vacuum concentration and purified using thin layer chromatography (DCM:MeOH = 100:1–20:1) to obtain a pale yellow viscous solid **8–11**.

**8a**, pale yellow viscous solid, yield 71%. ^1^H NMR (500 MHz, CDCl_3_) δ 7.38–7.32 (m, 5H), 7.31–7.27 (m, 5H), 7.20 (s, 1H), 6.71 (s, 1H), 6.48 (d, *J* = 7.0 Hz, 1H), 5.14 (s, 2H), 5.12 (s, 2H), 4.27 (t, *J* = 5.0 Hz, 2H), 4.04 (q, *J* = 5.0 Hz, 4H), 3.94 (d, *J* = 6.0 Hz, 2H), 2.62 (t, *J* = 6.0 Hz, 2H), 2.53 (t, *J* = 6.0 Hz, 2H), 2.40 (s, 1H), 2.11 (s, 3H). ^13^C NMR (125 MHz, CDCl_3_) δ 203.63, 173.49, 171.91, 170.14, 156.99, 146.09, 141.58, 139.25, 137.31, 136.61, 129.15, 128.57, 128.46, 128.25, 128.12, 128.08, 117.24, 73.33, 66.99, 62.18, 52.14, 49.03, 44.55, 34.15, 27.84, 12.76. ESI-HRMS: m/z calcd for C_30_H_33_N_3_O_8_Na [M + Na]^+^: 586.2160; found: 586.2149.

**8b**, pale yellow viscous solid, yield 55%. ^1^H NMR (500 MHz, CDCl_3_) δ 7.37 (d, *J* = 2.0 Hz, 1H), 7.36 (s, 1H), 7.32–7.28 (m, 5H), 7.27 (d, *J* = 2.0 Hz, 2H), 7.26–7.24 (m, 5H), 7.21 (s, 1H), 7.19 (t, *J* = 4.5 Hz, 2H), 6.45 (d, *J* = 7.5 Hz, 1H), 6.35 (d, *J* = 8.0 Hz, 1H), 5.17 (s, 2H), 5.03 (s, 2H), 4.54 (q, *J* = 7.5 Hz, 1H), 4.29–4.17 (m, 2H), 4.08 (dd, *J* = 19.0, 4.5 Hz, 1H), 4.02–3.94 (m, 3H), 3.21 (dd, *J* = 14.0, 5.5 Hz, 1H), 3.05 (dd, *J* = 13.5, 8.5 Hz, 1H), 2.62–2.56 (m, 2H), 2.55–2.46 (m, 2H), 2.11 (s, 3H). ^13^C NMR (125 MHz, CDCl_3_) δ 203.45, 173.39, 171.94, 171.86, 156.32, 146.08, 141.36, 139.14, 137.40, 137.05, 136.57, 129.49, 129.15, 128.61, 128.53, 128.44, 128.21, 128.04, 127.92, 126.85, 117.31, 73.30, 66.82, 62.25, 56.57, 52.07, 49.11, 38.45, 34.12, 27.75, 12.75. ESI-HRMS: m/z calcd for C_37_H_39_N_3_O_8_Na [M + Na]^+^: 676.2629; found: 676.2632.

**8c**, pale yellow viscous solid, yield 41%. ^1^H NMR (500 MHz, CDCl_3_) δ 7.38–7.32 (m, 5H), 7.31–7.28 (m, 5H), 7.27 (s, 1H), 7.21 (d, *J* = 7.5 Hz, 1H), 6.47 (d, *J* = 7.5 Hz, 1H), 6.35 (d, *J* = 8.0 Hz, 1H), 5.16 (s, 2H), 5.11 (s, 2H), 4.32–4.25 (m, 2H), 4.25–4.19 (m, 1H), 4.10 (dd, *J* = 19.5, 5.0 Hz, 1H), 4.05–3.97 (m, 3H), 2.65–2.60 (m, 2H), 2.58–2.47 (m, 2H), 2.11 (s, 3H), 1.80–1.65 (m, 2H), 1.63–1.57 (m, 1H), 0.94 (s, 3H), 0.93 (s, 3H). ^13^C NMR (125 MHz, CDCl_3_) δ 203.68, 203.64, 173.30, 171.90, 156.57, 146.04, 141.50, 139.22, 137.37, 136.63, 129.16, 128.56, 128.45, 128.23, 128.08, 128.04, 117.25, 73.32, 66.91, 62.23, 53.99, 52.15, 49.10, 41.54, 34.16, 27.77, 24.86, 23.22, 21.79, 12.77. ESI-HRMS: m/z calcd for C_34_H_41_N_3_O_8_Na [M + Na]^+^: 642.2786; found: 642.2792.

**9a**, pale yellow viscous solid, yield 42%. ^1^H NMR (500 MHz, CDCl_3_) δ 7.37–7.31 (m, 5H), 7.31–7.26 (m, 5H), 7.21 (d, *J* = 7.5 Hz, 1H), 7.15 (t, *J* = 5.0 Hz, 1H), 6.50 (t, *J* = 6.0 Hz, 1H), 6.42 (d, *J* = 7.5 Hz, 1H), 5.16 (s, 2H), 5.10 (s, 2H), 4.11–4.07 (m, 4H), 3.90 (d, *J* = 5.5 Hz, 2H), 3.76 (t, *J* = 7.5 Hz, 2H), 2.73–2.67 (m, 2H), 2.59–2.53 (m, 2H), 2.06 (s, 3H), 1.69–1.67 (m, 2H), 1.58–1.53 (m, 2H). ^13^C NMR (126 MHz, CDCl_3_) δ 203.72, 173.36, 172.52, 169.87, 157.00, 146.11, 141.21, 138.62, 137.47, 136.51, 129.24, 128.58, 128.39, 128.17, 128.14, 128.08, 117.26, 73.18, 67.04, 63.24, 53.25, 48.98, 44.58, 34.55, 27.75, 27.05, 25.64, 12.51. ESI-HRMS: m/z calcd for C_32_H_38_N_3_O_8_ [M + H]^+^: 592.2653; found: 592.2663.

**9b**, pale yellow viscous solid, yield 37%. ^1^H NMR (500 MHz, CDCl_3_) δ 7.37 (dd, *J* = 7.5, 2.0 Hz, 2H), 7.33–7.30 (m, 3H), 7.29–7.26 (m, 4H), 7.25 (s, 1H), 7.24–7.18 (m, 5H), 6.94 (t, *J* = 5.0 Hz, 1H), 6.46 (d, *J* = 7.5 Hz, 1H), 5.89 (d, *J* = 8.0 Hz, 1H), 5.19 (s, 2H), 5.04 (s, 2H), 4.50 (d, *J* = 7.5 Hz, 1H), 4.16–4.00 (m, 4H), 3.76 (td, *J* = 7.0, 2.0 Hz, 2H), 3.14 (dd, *J* = 14.0, 6.0 Hz, 1H), 3.07–3.04 (m, 1H), 2.67–2.65 (m, 2H), 2.56 (t, *J* = 6.0 Hz, 2H), 2.07 (s, 3H), 1.70–1.64 (m, 2H), 1.59–1.54 (m, 2H). ^13^C NMR (125 MHz, CDCl_3_) δ 203.54, 173.34, 172.47, 171.59, 156.24, 146.14, 141.02, 138.46, 137.57, 136.72, 136.39, 129.43, 129.28, 128.74, 128.60, 128.38, 128.18, 128.14, 128.02, 127.04, 117.37, 73.15, 67.03, 63.47, 56.47, 53.28, 49.12, 34.53, 29.81, 27.75, 27.18, 25.57, 12.55. ESI-HRMS: m/z calcd for C_39_H_44_N_3_O_8_ [M + H]^+^: 682.3132; found: 682.3125.

**9c**, pale yellow viscous solid, yield 46%. ^1^H NMR (500 MHz, CDCl_3_) δ 7.39–7.34 (m, 2H), 7.32 (d, *J* = 5.0 Hz, 3H), 7.32–7.27 (m, 5H), 7.20 (d, *J* = 7.5 Hz, 1H), 7.06 (t, *J* = 5.0 Hz, 1H), 6.44 (d, *J* = 7.5 Hz, 1H), 6.00 (d, *J* = 8.0 Hz, 1H), 5.18 (s, 2H), 5.10 (s, 2H), 4.28–4.30 (m, 1H), 4.16–4.06 (m, 4H), 3.81–3.72 (m, 2H), 2.71 (q, *J* = 5.0 Hz, 2H), 2.58 (d, *J* = 6.0 Hz, 2H), 2.06 (s, 3H), 1.71–1.64 (m, 4H), 1.61–1.53 (m, 3H), 0.93 (s, 3H), 0.92 (s, 3H). ^13^C NMR (125 MHz, CDCl_3_) δ 203.75, 173.30, 172.99, 172.50, 156.51, 146.01, 141.00, 138.47, 137.56, 136.49, 129.26, 128.59, 128.37, 128.16, 128.13, 128.07, 117.34, 73.13, 67.02, 63.40, 53.89, 53.26, 49.11, 41.44, 34.57, 27.74, 27.15, 25.59, 24.83, 23.16, 21.83, 12.53. ESI-HRMS: m/z calcd for C_36_H_46_N_3_O_8_ [M + H]^+^: 648.3279; found: 648.3275.

**10a**, pale yellow viscous solid, yield 45%. ^1^H NMR (500 MHz, CDCl_3_) δ 7.37 (d, *J* = 6.0 Hz, 2H), 7.32 (d, *J* = 5.0 Hz, 4H), 7.29 (d, *J* = 6.0 Hz, 4H), 7.21 (d, *J* = 7.0 Hz, 1H), 7.17 (t, *J* = 5.0 Hz, 1H), 6.44 (d, *J* = 7.0 Hz, 1H), 6.03 (s, 1H), 5.17 (s, 2H), 5.10 (s, 2H), 4.13 (d, *J* = 4.0 Hz, 2H), 4.04 (t, *J* = 6.5 Hz, 2H), 3.90 (d, *J* = 5.0 Hz, 2H), 3.74 (t, *J* = 7.0 Hz, 2H), 2.70 (t, *J* = 6.0 Hz, 2H), 2.58 (t, *J* = 6.0 Hz, 2H), 2.07 (s, 3H), 1.64–1.56 (m, 4H), 1.37–1.30 (m, 2H), 1.28–1.24 (m, 2H). ^13^C NMR (125 MHz, CDCl_3_) δ 203.93, 173.21, 172.54, 169.57, 156.83, 146.10, 141.19, 138.53, 137.54, 136.39, 129.23, 128.61, 128.37, 128.27, 128.23, 128.14, 117.21, 73.16, 67.16, 64.47, 53.80, 49.07, 44.52, 34.52, 30.57, 28.35, 27.83, 25.80, 25.52, 12.56. ESI-HRMS: m/z calcd for C_34_H_42_N_3_O_8_ [M + H]^+^: 620.2966; found: 620.2967.

**10b**, pale yellow viscous solid, yield 39%. ^1^H NMR (500 MHz, CDCl_3_) δ 7.38 (dd, *J* = 7.5, 2.0 Hz, 2H), 7.33–7.28 (m, 7H), 7.27 (d, *J* = 1.5 Hz, 1H), 7.25 (d, *J* = 7.5 Hz, 2H), 7.21 (d, *J* = 7.5 Hz, 2H), 7.18 (d, *J* = 7.5 Hz, 2H), 6.93 (t, *J* = 5.5 Hz, 1H), 6.49 (d, *J* = 7.5 Hz, 1H), 5.60 (d, *J* = 6.8 Hz, 1H), 5.19 (s, 2H), 5.08–5.01 (m, 2H), 4.49 (d, *J* = 7.5 Hz, 1H), 4.18–4.06 (m, 2H), 4.04 (t, *J* = 6.5 Hz, 2H), 3.75 (t, *J* = 7.5 Hz, 2H), 3.16–3.02 (m, 2H), 2.68–2.64 (m, 2H), 2.60–2.55 (m, 2H), 2.09 (s, 3H), 1.65–1.58 (m, 4H), 1.37–1.24 (m, 4H). ^13^C NMR (125 MHz, CDCl_3_) δ 203.69, 173.01, 172.53, 171.39, 156.12, 146.06, 141.30, 138.52, 137.55, 136.52, 136.31, 129.41, 129.26, 128.76, 128.62, 128.38, 128.23, 128.16, 128.07, 127.10, 117.17, 73.21, 67.10, 64.51, 53.93, 49.16, 38.55, 34.51, 30.64, 29.81, 28.39, 27.83, 25.91, 25.58, 12.59. ESI-HRMS: m/z calcd for C_41_H_48_N_3_O_8_ [M + H]^+^: 710.3436; found: 710.3434.

**10c**, pale yellow viscous solid, yield 42%. ^1^H NMR (500 MHz, CDCl_3_) δ 7.32 (dd, *J* = 7.5, 2.0 Hz, 2H), 7.27 (d, *J* = 4.5 Hz, 4H), 7.24 (d, *J* = 6.0 Hz, 4H), 7.21 (s, 1H), 6.93 (t, *J* = 4.5 Hz, 1H), 6.50 (d, *J* = 7.0 Hz, 1H), 5.45 (d, *J* = 8.0 Hz, 1H), 5.14 (s, 2H), 5.03 (s, 2H), 4.23–4.15 (m, 1H), 4.08 (s, 2H), 3.99 (t, *J* = 6.5 Hz, 2H), 3.72 (t, *J* = 7.5 Hz, 2H), 2.65 (t, *J* = 6.5 Hz, 2H), 2.54 (t, *J* = 6.5 Hz, 2H), 2.04 (s, 3H), 1.63–1.52 (m, 6H), 1.46 (t, *J* = 11.0 Hz, 1H), 1.33–1.27 (m, 2H), 1.23–1.18 (m, 2H), 0.86 (s, 3H), 0.85 (s, 3H). ^13^C NMR (125 MHz, CDCl_3_) δ 203.89, 172.70, 172.63, 172.56, 156.39, 145.95, 141.55, 138.63, 137.50, 136.38, 129.28, 128.65, 128.42, 128.26, 128.21, 128.15, 117.06, 73.30, 67.16, 64.50, 54.05, 53.74, 49.20, 41.65, 34.60, 30.63, 29.83, 28.40, 27.85, 25.88, 25.57, 24.84, 23.13, 12.65. ESI-HRMS: m/z calcd for C_38_H_50_N_3_O_8_ [M + H]^+^: 676.3592; found: 676.3584.

**11a**, pale yellow viscous solid, yield 39%. ^1^H NMR (500 MHz, CDCl_3_) δ 7.36 (dd, *J* = 7.5, 2.0 Hz, 2H), 7.31 (d, *J* = 5.0 Hz, 3H), 7.28–7.226 (m, 4H), 7.24 (t, *J* = 7.5 Hz, 1H), 7.17 (d, *J* = 7.5 Hz, 1H), 6.41 (d, *J* = 7.5 Hz, 1H), 6.02 (t, *J* = 5.5 Hz, 1H), 5.16 (s, 2H), 5.09 (s, 2H), 4.12 (d, *J* = 4.5 Hz, 2H), 4.02 (t, *J* = 6.5 Hz, 2H), 3.89 (d, *J* = 5.5 Hz, 2H), 3.71 (t, *J* = 7.5 Hz, 2H), 3.44 (s, 1H), 2.68 (t, *J* = 6.5 Hz, 2H), 2.58 (t, *J* = 6.5 Hz, 2H), 2.05 (s, 3H), 1.62–1.55 (m, 4H), 1.28–1.23 (m, 8H). ^13^C NMR (125 MHz, CDCl_3_) δ 203.97, 173.30, 172.56, 169.56, 156.77, 146.77, 141.07, 138.41, 137.53, 136.36, 129.18, 128.56, 128.31, 128.17, 128.09, 128.08, 117.19, 73.08, 67.10, 64.83, 53.92, 50.67, 49.06, 44.44, 34.49, 30.64, 28.95, 28.90, 28.45, 27.81, 26.17, 25.70, 12.47. ESI-HRMS: m/z calcd for C_36_H_46_N_3_O_8_ [M + H]^+^: 648.3279; found: 648.3275.

**11b**, pale yellow viscous solid, yield 34%. ^1^H NMR (500 MHz, CDCl_3_) δ 7.41–7.37 (m, 2H), 7.34–7.28 (m, 9H), 7.24–7.17 (m, 6H), 6.87 (s, 1H), 6.47 (d, *J* = 7.5 Hz, 1H), 5.54 (d, *J* = 6.5 Hz, 1H), 5.21 (s, 2H), 5.10–5.01 (m, 2H), 4.54–4.46 (m, 1H), 4.18–4.03 (m, 4H), 3.74 (t, *J* = 7.5 Hz, 2H), 3.14–3.10 (m, 2H), 2.66 (t, *J* = 6.0 Hz, 2H), 2.59 (t, *J* = 6.0 Hz, 2H), 2.09 (s, 3H), 1.64–1.58 (m, 4H), 1.33–1.27 (m, 8H). ^13^C NMR (125 MHz, CDCl_3_) δ 203.59, 173.12, 172.54, 171.29, 156.09, 146.11, 141.10, 138.41, 137.63, 136.48, 136.30, 129.39, 129.25, 128.77, 128.62, 128.37, 128.24, 128.12, 128.09, 127.12, 117.23, 73.16, 67.11, 64.90, 56.27, 54.03, 49.18, 38.57, 34.54, 30.73, 29.81, 29.04, 28.53, 27.86, 26.25, 25.77, 12.56. ESI-HRMS: m/z calcd for C_43_H_52_N_3_O_8_ [M + H]^+^: 738.3749; found: 738.3747.

**11c**, pale yellow viscous solid, yield 36%. ^1^H NMR (500 MHz, CDCl_3_) δ 7.39–7.36 (m, 2H), 7.34–7.32 (m, 4H), 7.30–7.27 (m, 4H), 7.21 (d, *J* = 7.5 Hz, 1H), 7.02 (s, t, *J* = 4.5 Hz, 1H), 6.47 (d, *J* = 7.5 Hz, 1H), 5.52 (d, *J* = 8.0 Hz, 1H), 5.19 (s, 2H), 5.09 (d, *J* = 4.0 Hz, 2H), 4.17–4.11 (m, 2H), 4.04 (t, *J* = 6.5 Hz, 2H), 3.74 (t, *J* = 7.5 Hz, 2H), 2.74–2.68 (m, 2H), 2.60 (t, *J* = 6.5 Hz, 2H), 2.08 (s, 3H), 1.91–1.89 (m, 1H), 1.70–1.65 (m, 3H), 1.63–1.56 (m, 5H), 1.54–1.49 (m, 1H), 1.29 (s, 6H), 0.92 (s, 3H), 0.91 (s, 3H). ^13^C NMR (125 MHz, CDCl_3_) δ 203.85, 173.03, 172.68, 172.56, 157.09, 156.35, 146.07, 141.20, 138.45, 137.57, 136.36, 129.23, 128.61, 128.36, 128.23, 128.12, 117.19, 73.17, 67.12, 64.88, 54.06, 49.19, 41.68, 34.59, 34.05, 30.72, 29.02, 28.52, 27.85, 26.24, 25.76, 25.08, 24.80, 23.10, 21.94, 12.55. ESI-HRMS: m/z calcd for C_40_H_53_N_3_O_8_ [M + H]^+^: 726.3725; found: 726.3725.

#### General procedure for the preparation of compounds AP-1–12

To a 100 ml round-bottom flask was added intermediates **8-11a** (0.2 mmol) and anhydrous DCM (10 ml) sequentially in an atmosphere of N_2_. The resulting mixture was stirred at −40 °C for 30 min and then the dry DCM solution (10 ml) of BBr_3_ (0.4 mmol) was added dropwise through a pressure-equalizing dropping funnel. Upon completion of the addition, the resulting mixture was naturally heated up to room temperature and continuously stirred at room temperature for 4–8 h and monitored by TLC. After the reaction was completed, the mixture was quenched with MeOH and subjected to vacuum concentration to obtain yellow oily crude products (**AP-1–4**).

To a 100 ml round-bottom flask was added intermediates **8-11b-c** (M mg, 0.2 mmol), Pd/C (0.2–0.4*M), benzyl chloride (0.44 mmol) and anhydrous MeOH (5 ml) sequentially in the atmosphere of 30 psi H_2_. The resulting mixture was stirred at room temperature for 1–6 h and monitored by TLC. After the reaction was completed, the mixture was filtered and the filtrate was subjected to vacuum concentration to obtain yellow oily crude products (**AP-5–12**).

The crude products were purified by preparative Waters 2535Q HPLC using reverse-phase Waters SunFire Prep C18 OBD column (19 mm × 250 mm) eluted with acetonitrile/water (25:75, V/V) with a flow rate of 5 ml/min at wavelength 264 nm. The collected product solution was subjected to vacuum concentration, and then recrystallized with methanol/ether to obtain a viscous oily substance. The substances were then purified by a high-quality vacuum pump to remove residual solvents, resulting in viscous solids (**AP-1–12**).

##### 2–(3-hydroxy-2-methyl-4-oxopyridin-1(4*H*)-yl)ethyl 5–(2-aminoacetamido)-4-oxopentanoate (AP-1)

Brown viscous solid, yield 65%. ^1^H NMR (500 MHz, DMSO-*d*_6_) δ 8.62 (t, *J* = 5.0 Hz, 1H), 8.20 (d, *J* = 7.0 Hz, 1H), 8.01 (s, 2H), 7.15 (d, *J* = 7.0 Hz, 1H), 4.63 (t, *J* = 4.5 Hz, 2H), 4.39 (t, *J* = 5.0 Hz, 2H), 4.10 (t, *J* = 5.0 Hz, 2H), 3.64–3.60 (m, 3H), 3.57 (s, 1H), 2.75–2.66 (m, 2H), 2.56 (s, 3H). ^13^C NMR (125 MHz, DMSO-*d*_6_) δ 204.61, 172.64, 166.20, 158.20, 142.65, 142.36, 139.05, 110.31, 59.53, 58.03, 51.45, 48.29, 34.03, 27.18, 12.85. ESI-HRMS: m/z calcd for C_15_H_22_N_3_O_6_ [M + H]^+^: 340.1503; found: 340.1505.

##### 4–(3-hydroxy-2-methyl-4-oxopyridin-1(4*H*)-yl)butyl 5–(2-aminoacetamido)-4-oxopentanoate (AP-2)

Brown viscous solid, yield 64%. ^1^H NMR (500 MHz, DMSO-*d*_6_) δ 10.55 (s, 1H), 8.42 (s, 1H), 8.25 (d, *J* = 7.0 Hz, 1H), 7.99 (s, 1H), 7.14 (d, *J* = 7.0 Hz, 1H), 4.36 (t, *J* = 7.5 Hz, 3H), 4.12 (d, *J* = 5.1 Hz, 1H), 3.60 (s, 1H), 3.58 (s, 1H), 3.43 (t, *J* = 6.5 Hz, 2H), 2.83–2.77 (m, 1H), 2.77–2.71 (m, 1H), 2.55 (s, 3H), 2.50 (s, 2H), 1.85–1.74 (m, 2H), 1.48–1.42 (m, 2H). ^13^C NMR (125 MHz, DMSO-*d*_6_) δ 204.60, 172.63, 166.19, 158.04, 142.94, 141.75, 138.19, 110.68, 59.95, 55.96, 51.44, 48.29, 34.16, 34.03, 28.78, 26.50, 12.49. ESI-HRMS: m/z calcd for C_17_H_26_N_3_O_6_ [M + H]^+^: 368.1816; found: 368.1824.

##### 6–(3-hydroxy-2-methyl-4-oxopyridin-1(4*H*)-yl)hexyl 5–(2-aminoacetamido)-4-oxopentanoate (AP-3)

Brown viscous solid, yield 61%. ^1^H NMR (500 MHz, DMSO-*d*_6_) δ 10.52 (s, 1H), 8.67–8.60 (m, 1H), 8.28 (d, *J* = 7.0 Hz, 1H), 8.01 (s, 3H), 7.18 (d, *J* = 7.0 Hz, 1H), 4.34 (t, *J* = 7.5 Hz, 2H), 4.10 (t, *J* = 4.0 Hz, 2H), 3.98 (t, *J* = 7.0 Hz, 2H), 3.63 (q, *J* = 6.0 Hz, 2H), 2.73 (t, *J* = 6.0 Hz, 2H), 2.68 (t, *J* = 7.0 Hz, 1H), 2.55 (s, 3H), 2.43 (t, *J* = 7.0 Hz, 1H), 1.74 (t, *J* = 7.5 Hz, 2H), 1.58–1.52 (m, 2H), 1.39–1.25 (m, 4H). ^13^C NMR (125 MHz, DMSO-*d*_6_) δ 204.73, 173.58, 166.18, 158.07, 142.93, 141.72, 138.18, 110.68, 63.86, 55.92, 48.34, 34.13, 29.40, 27.89, 27.47, 27.31, 25.19, 24.84, 12.50. ESI-HRMS: m/z calcd for C_19_H_30_N_3_O_6_ [M + H]^+^: 396.2129; found: 396.2134.

##### 8–(3-hydroxy-2-methyl-4-oxopyridin-1(4*H*)-yl)octyl 5–(2-aminoacetamido)-4-oxopentanoate (AP-4)

Brown viscous solid, yield 60%. ^1^H NMR (500 MHz, DMSO-*d*_6_) δ 10.54 (s, 1H), 8.68 (q, *J* = 5.0 Hz, 1H), 8.36–8.30 (m, 1H), 8.06 (s, 2H), 7.22 (dd, *J* = 7.0, 3.0 Hz, 1H), 4.36 (t, *J* = 7.5 Hz, 2H), 4.12–4.10 (m, 2H), 4.02–3.97 (m, 1H), 3.64 (q, *J* = 5.5 Hz, 2H), 3.58 (s, 1H), 3.53 (t, *J* = 7.0 Hz, 1H), 3.37 (t, *J* = 7.0 Hz, 1H), 2.75 (q, *J* = 6.5 Hz, 1H), 2.69 (t, *J* = 6.5 Hz, 1H), 2.56 (s, 3H), 2.44 (t, *J* = 6.5 Hz, 2H), 1.77–1.68 (m, 2H), 1.40–1.30 (m, 8H). ^13^C NMR (125 MHz, DMSO-*d*_6_) δ 204.61, 172.62, 166.16, 158.01, 142.90, 141.75, 138.19, 110.66, 63.96, 60.64, 56.00, 51.44, 48.29, 35.24, 34.03, 32.14, 29.53, 27.87, 27.37, 25.23, 12.52. ESI-HRMS: m/z calcd for C_21_H_34_N_3_O_6_ [M + H]^+^: 424.2442; found: 424.2454.

##### 2–(3-hydroxy-2-methyl-4-oxopyridin-1(4*H*)-yl)ethyl (*S*)-5–(2-amino-3-phenylpropanamido)-4-oxopentanoate (AP-5)

Yellow viscous solid, yield 60%. ^1^H NMR (500 MHz, DMSO-*d*_6_) δ 9.07 (t, *J* = 5.5 Hz, 1H), 8.38 (s, 3H), 8.19 (d, *J* = 7.0 Hz, 1H), 7.35 (d, *J* = 7.0 Hz, 1H), 7.33–7.30 (m, 5H), 4.66–4.57 (m, 2H), 4.41–4.37 (m, 2H), 3.99 (d, *J* = 7.0 Hz, 2H), 3.57 (s, 1H), 3.17–3.13 (m, 1H), 3.09–3.04 (m, 1H), 2.63 (q, *J* = 6.0 Hz, 2H), 2.54 (s, 3H), 2.45 (t, *J* = 6.5 Hz, 2H). ^13^C NMR (125 MHz, DMSO-*d*_6_) δ 204.72, 172.60, 171.79, 168.38, 143.03, 138.86, 134.98, 129.61, 128.46, 128.26, 127.08, 110.59, 61.96, 53.25, 51.42, 48.27, 36.89, 33.82, 27.10, 12.69. ESI-HRMS: m/z calcd for C_22_H_28_N_3_O_6_ [M + H]^+^: 430.1973; found: 430.1990.

##### 4–(3-hydroxy-2-methyl-4-oxopyridin-1(4*H*)-yl)butyl (*S*)-5–(2-amino-3-phenylpropanamido)-4-oxopentanoate (AP-6)

Yellow viscous solid, yield 58%. ^1^H NMR (500 MHz, DMSO-*d*_6_) δ 9.06 (t, *J* = 5.5 Hz, 1H), 8.37 (s, 2H), 8.28 (d, *J* = 7.0 Hz, 1H), 7.39 (d, *J* = 7.0 Hz, 1H), 7.31 (d, *J* = 4.5 Hz, 3H), 7.30–7.25 (m, 2H), 4.36 (d, *J* = 7.5 Hz, 2H), 4.08–3.92 (m, 4H), 3.45–3.35 (m, 1H), 3.14–3.07 (m, 2H), 2.67 (d, *J* = 4.5 Hz, 2H), 2.54 (s, 3H), 2.48 (d, *J* = 6.5 Hz, 2H), 1.83–1.74 (m, 2H), 1.64–1.59 (m, 2H). ^13^C NMR (125 MHz, DMSO-*d*_6_) δ 204.84, 172.15, 168.36, 158.48, 142.96, 141.44, 138.03, 134.97, 129.60, 128.46, 127.09, 110.77, 63.30, 59.93, 55.45, 53.26, 48.31, 36.92, 33.95, 26.15, 24.84, 12.45. ESI-HRMS: m/z calcd for C_24_H_32_N_3_O_6_ [M + H]^+^: 458.2286; found: 458.2278.

##### 6–(3-hydroxy-2-methyl-4-oxopyridin-1(4*H*)-yl)hexyl (*S*)-5–(2-amino-3-phenylpropanamido)-4-oxopentanoate (AP-7)

Yellow viscous solid, yield 57%. ^1^H NMR (500 MHz, DMSO-*d*_6_) δ 10.50 (s, 1H), 9.05 (t, *J* = 5.5 Hz, 1H), 8.37 (s, 1H), 8.26 (d, *J* = 6.0 Hz, 1H), 7.38 (s, 1H), 7.30–7.14 (m, 5H), 4.31 (t, *J* = 8.0 Hz, 2H), 4.14 (s, 1H), 4.07–3.97 (m, 2H), 3.62–3.32 (m, 2H), 3.16–3.01 (m, 2H), 2.70–2.61 (m, 2H), 2.53 (s, 3H), 2.49–2.45 (m, 2H), 1.73 (t, *J* = 8.0 Hz, 2H), 1.55 (q, *J* = 7.0 Hz, 2H), 1.35–1.25 (m, 4H). ^13^C NMR (125 MHz, DMSO-*d*_6_) δ 204.78, 172.15, 168.33, 158.50, 142.94, 141.28, 137.99, 134.97, 129.59, 128.45, 127.07, 110.71, 64.91, 63.83, 60.46, 55.79, 53.25, 48.31, 36.90, 33.95, 29.39, 24.84, 15.16, 12.44. ESI-HRMS: m/z calcd for C_26_H_36_N_3_O_6_ [M + H]^+^: 486.2599; found: 486.2639.

##### 8–(3-hydroxy-2-methyl-4-oxopyridin-1(4*H*)-yl)octyl (*S*)-5–(2-amino-3-phenylpropanamido)-4-oxopentanoate (AP-8)

Yellow viscous solid, yield 55%. ^1^H NMR (500 MHz, DMSO-*d*_6_) δ 9.03 (t, *J* = 5.5 Hz, 1H), 8.36 (s, 1H), 8.25 (d, *J* = 6.0 Hz, 1H), 7.35 (t, *J* = 6.0 Hz, 1H), 7.33–7.04 (m, 5H), 4.29 (d, *J* = 8.0 Hz, 2H), 4.20–3.64 (m, 4H), 3.61–3.29 (m, 1H), 3.31–2.88 (m, 2H), 2.78–2.55 (m, 2H), 2.52 (s, 3H), 2.47–2.33 (m, 2H), 1.71 (s, 2H), 1.61–1.39 (m, 2H), 1.30–1.21 (m, 8H). ^13^C NMR (125 MHz, DMSO-*d*_6_) δ 204.74, 172.11, 168.32, 158.62, 142.97, 141.05, 137.97, 134.95, 129.58, 128.44, 127.06, 110.70, 63.89, 60.63, 55.81, 53.24, 48.30, 45.27, 36.90, 33.94, 29.50, 28.43, 28.02, 25.50, 25.20, 12.41. ESI-HRMS: m/z calcd for C_28_H_40_N_3_O_6_ [M + H]^+^: 514.2912; found: 514.2939.

##### 2–(3-hydroxy-2-methyl-4-oxopyridin-1(4*H*)-yl)ethyl (*S*)-5–(2-amino-4-methylpentanamido)-4-oxopentanoate (AP-9)

Yellow viscous solid, yield 62%. ^1^H NMR (500 MHz, DMSO-*d*_6_) δ 9.03 (t, *J* = 5.5 Hz, 1H), 8.40 (s, 3H), 8.20 (d, *J* = 7.0 Hz, 1H), 7.37 (d, *J* = 7.0 Hz, 1H), 4.62 (t, *J* = 5.0 Hz, 2H), 4.39 (t, *J* = 5.0 Hz, 2H), 4.10 (dd, *J* = 18.5, 6.0 Hz, 1H), 3.97 (dd, *J* = 18.0, 5.0 Hz, 1H), 3.84 (s, 1H), 2.72 (t, *J* = 6.5 Hz, 2H), 2.54 (s, 3H), 2.47 (d, *J* = 7.0 Hz, 2H), 1.73 (dt, *J* = 13.5, 6.5 Hz, 1H), 1.59 (t, *J* = 7.0 Hz, 2H), 0.90 (d, *J* = 6.5 Hz, 3H), 0.89 (d, *J* = 6.5 Hz, 3H). ^13^C NMR (125 MHz, DMSO-*d*_6_) δ 204.57, 171.82, 169.33, 159.59, 143.02, 141.11, 138.86, 110.57, 61.97, 54.35, 50.72, 48.21, 40.31, 33.93, 27.12, 23.53, 22.50, 22.28, 12.70. ESI-HRMS: m/z calcd for C_19_H_30_N_3_O_6_ [M + H]^+^: 396.2129; found: 396.2125.

##### 4–(3-hydroxy-2-methyl-4-oxopyridin-1(4*H*)-yl)butyl (*S*)-5–(2-amino-4-methylpentanamido)-4-oxopentanoate (AP-10)

Yellow viscous solid, yield 60%. ^1^H NMR (500 MHz, DMSO-*d*_6_) δ 10.53 (s, 1H), 9.02 (t, *J* = 5.5 Hz, 1H), 8.39 (d, *J* = 5.5 Hz, 2H), 8.29 (dd, *J* = 7.0, 3.0 Hz, 1H), 7.41 (dd, *J* = 10.0, 7.0 Hz, 1H), 4.36 (dt, *J* = 9.5, 7.5 Hz, 2H), 4.14–4.11 (m, 1H), 4.03 (t, *J* = 6.5 Hz, 2H), 3.90–3.82 (m, 1H), 2.74 (dd, *J* = 7.5, 6.0 Hz, 2H), 2.54 (s, 3H), 2.52 (d, *J* = 4.0 Hz, 1H), 2.45 (d, *J* = 7.5 Hz, 1H), 1.82–1.75 (m, 4H), 1.63–1.57 (m, 4H), 0.90 (d, *J* = 6.5 Hz, 3H), 0.89 (d, *J* = 6.5 Hz, 3H). ^13^C NMR (125 MHz, DMSO-*d*_6_) δ 204.67, 172.19, 169.30, 158.55, 142.96, 141.41, 138.04, 110.77, 63.31, 59.92, 55.44, 50.72, 48.25, 34.05, 27.31, 26.16, 24.84, 23.53, 22.50, 22.25, 12.47. ESI-HRMS: m/z calcd for C_21_H_34_N_3_O_6_ [M + H]^+^: 424.2442; found: 424.2462.

##### 6–(3-hydroxy-2-methyl-4-oxopyridin-1(4*H*)-yl)hexyl (*S*)-5–(2-amino-4-methylpentanamido)-4-oxopentanoate (AP-11)

Yellow viscous solid, yield 59%. ^1^H NMR (500 MHz, DMSO-*d*_6_) δ 10.52 (s, 1H), 9.01 (t, *J* = 5.5 Hz, 1H), 8.43–8.35 (m, 2H), 8.28 (d, *J* = 7.0 Hz, 1H), 7.41 (d, *J* = 7.0 Hz, 1H), 4.37–4.28 (m, 2H), 4.12 (dd, *J* = 18.0, 6.0 Hz, 1H), 4.02–3.95 (m, 2H), 3.83 (q, *J* = 6.5 Hz, 1H), 2.73 (t, *J* = 6.5 Hz, 2H), 2.54 (s, 3H), 2.49–2.46 (m, 2H), 1.78–1.67 (m, 3H), 1.59–1.55 (m, 4H), 1.39–1.18 (m, 5H), 0.90 (d, *J* = 6.5 Hz, 3H), 0.89 (d, *J* = 6.5 Hz, 3H). ^13^C NMR (125 MHz, DMSO-*d*_6_) δ 204.61, 172.19, 169.28, 158.48, 142.93, 141.34, 138.01, 110.71, 63.84, 55.81, 50.72, 48.25, 40.32, 34.04, 29.39, 27.87, 27.30, 25.19, 24.85, 23.52, 22.49, 22.25, 12.47. ESI-HRMS: m/z calcd for C_23_H_38_N_3_O_6_ [M + H]^+^: 452.2755; found: 452.2750.

##### 8–(3-hydroxy-2-methyl-4-oxopyridin-1(4*H*)-yl)octyl (*S*)-5–(2-amino-4-methylpentanamido)-4-oxopentanoate (AP-12)

Yellow viscous solid, yield 58%. ^1^H NMR (500 MHz, DMSO-*d*_6_) δ 10.52 (s, 1H), 9.05–8.86 (m, 1H), 8.37 (s, 2H), 8.27 (d, *J* = 7.0 Hz, 1H), 7.40 (d, *J* = 7.0 Hz, 1H), 4.32 (t, *J* = 7.5 Hz, 2H), 4.13 (dd, *J* = 18.5, 6.0 Hz, 1H), 4.05–3.91 (m, 3H), 3.83 (s, 1H), 2.73 (t, *J* = 6.5 Hz, 2H), 2.53 (s, 3H), 1.80–1.66 (m, 3H), 1.64–1.50 (m, 4H), 1.32–1.28 (m, 10H), 0.90 (d, *J* = 6.5 Hz, 3H), 0.89 (d, *J* = 6.5 Hz, 3H). ^13^C NMR (125 MHz, DMSO-*d*_6_) δ 204.56, 172.19, 169.27, 158.44, 142.92, 141.35, 138.00, 110.71, 63.93, 55.89, 50.71, 48.24, 40.32, 34.04, 29.50, 28.43, 28.33, 28.02, 27.29, 25.51, 25.20, 23.51, 22.49, 22.22, 12.45. ESI-HRMS: m/z calcd for C_25_H_42_N_3_O_6_ [M + H]^+^: 480.3068; found: 480.3076.

### Dark cytotoxicity assay

The dark cytotoxicity of compounds **AP-1–12** was determined using A375 (Art.No. JCM-H1015) and MCF-7 (Art.No. JCM-H1165) cell lines, which were purchased from Hangzhou JCM Biotechnology Co., Ltd.(www.jcellm.com). Cells were authenticated by STR profiling and tested negative for mycoplasma contamination. ALA and ALA+DFP were used as positive controls. The cells were cultured in a DMEM high-glucose medium containing 10% foetal bovine serum. Cells in the logarithmic growth phase were digested, counted, and seeded at a density of 8000 cells/well in a 96-well plate. Following a 24-h incubation at 37 °C in a 5% CO_2_, the original culture medium was discarded and replaced with solutions of the compounds at various concentrations (10, 50, 100, 200 µM) prepared in serum-free culture medium. The 96-well plates were then covered with aluminium foil and incubated in the dark for an additional 4 h. Subsequently, the drug-containing medium was removed and replaced with 100 μL of fresh serum-free medium, and the cultures were further incubated for 18 h. Finally, 10 μL of CCK-8 solution was added, and after an additional 2 h of incubation, the absorbance was measured at 450 nm. Cell viability was calculated using the following formula, with the untreated cell wells serving as controls and PBS used as blanks.

$$\text{Cell viability}\;(\%)=\frac{{\text{OD}}_{\text{Sample}}-{\text{OD}}_{\text{Blank}}}{{\text{OD}}_{\text{Control}}-{\text{OD}}_{\text{Blank}}}\times 100\%$$

### Cellular phototoxicity assay

The phototoxicity of compound **AP-1–12** was determined using A375 and MCF-7 cell lines. ALA and ALA+DFP were used as positive controls. The cells were cultured in a DMEM high-glucose medium containing 10% foetal bovine serum (purchased from OmnimAbs, origin: Australia Excellent). Cells in the logarithmic growth phase were digested, counted, and seeded at a density of 8000 cells/well in a 96-well plate. Following a 24-h incubation at 37 °C in a 5% CO_2_, the original culture medium was discarded and replaced with solutions of the compounds at various concentrations (1, 5, 10, 20, 22, 24, 26, 28, 30, 35, 40, 50, 80, 100, 150, 200, 400, 800,1000, 2000 µM) prepared in serum-free culture medium. The 96-well plates were then covered with aluminium foil and incubated in the dark for an additional 4 h. Subsequently, the plates were exposed to blue light (∼420 nm, light dose 5 J/cm^2^) for 5 min. Immediately after irradiation, the drug-containing medium was removed and replaced with 100 μL of fresh serum-free medium, and the cultures were further incubated for 18 h. Finally, 10 μL of CCK-8 solution was added, and after an additional 2 h of incubation, the absorbance was measured at 450 nm. Cell inhibitory rate was calculated using the following formula, with the untreated cell wells serving as controls and PBS used as blanks. The IC_50_ value was calculated using GraphPad Prism 8.0.

$$\text{Cell inhibitory}\;(\%)=\frac{{\text{OD}}_{\text{Control}}-{\text{OD}}_{\text{Sample}}}{{\text{OD}}_{\text{Control}}-{\text{OD}}_{\text{Blank}}}$$

### PpIX fluorescence kinetics assay

The PpIX fluorescence kinetics of two selected compounds (**AP-8** and **AP-12**) were investigated using A375 and MCF-7 cell lines. The cells were cultured in a DMEM high-glucose medium containing 10% foetal bovine serum. Cells in the logarithmic growth phase were digested, counted, and seeded at a density of 8000 cells/well in a 96-well plate. Following a 24-h incubation at 37 °C in a 5% CO_2_, the original culture medium was discarded and replaced with solutions of the compounds at various concentrations (10, 50, 100, 200 µM) prepared in serum-free culture medium. The 96-well plates were then covered with aluminium foil and incubated in the dark for various times (2, 4, 6, 8, 12, 24 h). Subsequently, the drug-containing culture medium was removed, and 50 μL of RIPA lysis buffer was added. After shaking on a horizontal shaker for 5 min, the fluorescence intensity of each well was measured using a microplate reader (excitation wavelength 405 nm, emission wavelength 635 nm).

### *In vivo* photodynamic anti-tumour assay

This experiment has received ethical approval from the Animal Ethical and Welfare Committee of Hangzhou Normal University (Approval No. HSD-20260304–01). The purpose is to evaluate the inhibitory effects of the compound **AP-8** and the positive drug ALA on melanoma in mice under irradiated or non-irradiated conditions. 48 male BALB/c nude mice (3–5 weeks, 20–22 g) were purchased from GemPharmatech Co., Ltd. And housed in specific pathogen‑free (SPF) conditions in an individually ventilated cage (IVC) system. In the right axilla of each mouse, a subcutaneous injection of 100 μL of a suspension containing A375 cells at a concentration of 5 × 10^7^ cells/mL was performed to establish a model of subcutaneous melanoma. When the tumour volume reached between 50–100 mm³, the mice were randomly divided into six groups (control non-irradiated, control irradiated, ALA non-irradiated, ALA irradiated, **AP-8** non-irradiated, **AP-8** irradiated), with 8 mice in each group. The compounds were solubilised in a physiological saline solution containing 10% DMSO and 5% Tween-80. Each group received an intratumoral injection of 50 μL of either the blank solvent or the compound at a dose of 15 mg/kg. The irradiated groups were subsequently exposed to MDL-III-635 laser light for 5 min, one hour post-administration, at a wavelength of 635 nm and a light dose of 180 J/cm^2^. The body weight and tumour volume of the mice were recorded daily. After 7 consecutive days of treatment, the mice were euthanized by cervical dislocation following isoflurane inhalation anaesthesia. And tumours were excised, weighed, and measured for volume. Tumour tissue samples were also collected for H&E staining analysis. Tumour volume was calculated using the formula: V = (longest diameter (L) × shortest diameter (W)2)/2 (mm^3^). Tumour growth inhibition (TGI) was calculated using the following formula: RTV represents the relative tumour volume.

$$\text{TGI}\;(\%)=[1-\frac{{\text{RTV}}_{\text{Treatment group}}}{{\text{RTV}}_{\text{Control group}}}]\times 100\%$$

## Data Availability

Data will be made available on request.
